# Gasdermin pores permeabilize mitochondria to augment caspase-3 activation during apoptosis and inflammasome activation

**DOI:** 10.1038/s41467-019-09397-2

**Published:** 2019-04-11

**Authors:** Corey Rogers, Dan A. Erkes, Alexandria Nardone, Andrew E. Aplin, Teresa Fernandes-Alnemri, Emad S. Alnemri

**Affiliations:** 1 Department of Biochemistry and Molecular Biology, Sidney Kimmel Cancer Center, Thomas Jefferson University, Philadelphia, PA 19107 USA; 2 Department of Cancer Biology, Sidney Kimmel Cancer Center, Thomas Jefferson University, Philadelphia, PA 19107 USA

## Abstract

Gasdermin E (GSDME/DFNA5) cleavage by caspase-3 liberates the GSDME-N domain, which mediates pyroptosis by forming pores in the plasma membrane. Here we show that GSDME-N also permeabilizes the mitochondrial membrane, releasing cytochrome c and activating the apoptosome. Cytochrome c release and caspase-3 activation in response to intrinsic and extrinsic apoptotic stimuli are significantly reduced in GSDME-deficient cells comparing with wild type cells. GSDME deficiency also accelerates cell growth in culture and in a mouse model of melanoma. Phosphomimetic mutation of the highly conserved phosphorylatable Thr6 residue of GSDME, inhibits its pore-forming activity, thus uncovering a potential mechanism by which GSDME might be regulated. Like GSDME-N, inflammasome-generated gasdermin D-N (GSDMD-N), can also permeabilize the mitochondria linking inflammasome activation to downstream activation of the apoptosome. Collectively, our results point to a role of gasdermin proteins in targeting the mitochondria to promote cytochrome c release to augment the mitochondrial apoptotic pathway.

## Abstract (web summary)

Gasdermins mediate lytic cell death by forming pores in the plasma membrane. Here the authors show that gasdermins also permeabilize mitochondrial membrane, thereby facilitating intrinsic apoptosis pathway, downstream of apoptotic (Gasdermin E) and inflammatory (Gasdermin D) caspase activation.

## Introduction

Apoptosis is a form of programmed cell death (PCD) that plays critical roles in embryonic development, maintenance and regulation of a healthy immune system, and tumor suppression. It is initiated in cells by a diverse range of physiological and pathological stimuli, which ultimately lead to activation of the intrinsic or extrinsic apoptotic pathways^1,2^. The intrinsic pathway is activated by internal stress arising from stimuli such as DNA damage, viral infection, glucocorticoids, and hypoxia leading to Bax/Bak-mediated pore formation on the outer mitochondrial membrane, which facilitates the release of proapoptotic proteins such as cytochrome *c* (Cyt *c*) and HtrA2/Omi into the cytosol^3,4^. Cytosolic Cyt *c* binds to Apaf-1 (apoptotic protease activating factor-1), leading to the recruitment and activation of procaspase-9. Active caspase-9 then cleaves and activates procaspase-3/7, which in turn leads to cellular demise by cleaving hundreds of different cellular substrates^2^.

The extrinsic pathway is activated when ligands such as tumor necrosis factor-α (TNFα) bind to death receptors^1,2^. The ensuing oligomerization of these receptors leads to recruitment and activation of caspase-8, which in turn directly cleaves procaspase-3 to mediate cellular dismantling. Interestingly, activation of this pathway can also activate the intrinsic pathway when caspase-8 cleaves the cytosolic Bcl-2 (B-cell lymphoma 2) family member Bid^5,6^. This cleavage generates a truncated fragment called tBid that translocates to the mitochondria where it activates Bax/Bak pores to release cytochrome *c* and activate the Apaf-1 apoptosome.

Pyroptosis is a necrotic form of PCD mediated by members of the gasdermin superfamily, which include GSDMA, GSDMB, GSDMC, GSDMD, and GSDME (or DFNA5)^7–12^. These proteins have been recently shown to possess intrinsic necrotic activity in their gasdermin-N domains that is normally masked by their gasdermin-C domains^9,12,13^. Proteolytic cleavage between their gasdermin-N and -C domains releases the inhibitory gasdermin-C domain allowing the necrotic gasdermin-N domain to translocate and form oligomers in the plasma membrane^9,12–16^. These oligomers form membrane-spanning pores that allow for the release of inflammatory molecules such as interleukin (IL)-1β, IL-18, and high-mobility group box 1 (HMGB1) as well as osmotic swelling leading to cytolysis^7–9^. Among the gasdermin proteins, only GSDMD and GSDME are cleaved by caspases between their gasdermin-N and -C domains to form membrane pores^7–12^. GSDME is cleaved by caspase-3 to induce pyroptosis downstream of apoptosis, whereas GSDMD is cleaved by inflammatory caspases to induce pyroptosis downstream of inflammasome activation. GSDMA, GSDMB, and GSDMC also possess pore-forming gasdermin-N domains^12^, but none of them have been shown to be cleaved in response to physiological or pathological stimuli to form functional pores.

In addition to their necrotic activity, GSDMA, GSDMC, GSDMD, and GSDME have all been proposed to possess tumor suppressive activity, as their expression suppresses cell proliferation and colony formation in gastric and colorectal cancer cell lines^17–20^. Furthermore, expression of GSDMA, GSDMC, and GSDMD was found to be downregulated in primary esophageal squamous cell carcinoma and gastric cancer tumors^19^, and expression of GSDME has been shown to be downregulated in breast, gastric, and colorectal cancers due to promoter hypermethylation^17,18,21,22^. In addition, reduced GSDME expression decreases sensitivity of cancer cell lines to etoposide-induced apoptosis, while its ectopic overexpression increases their sensitivity^23,24^. Lastly, GSDME expression is regulated by p53^24^, which is known to activate the transcription of numerous tumor suppressors and activators of apoptosis.

While the necrotic activity of gasdermins has recently been extensively characterized, their tumor suppressive activity is much less characterized as tumor suppressors typically act upstream of apoptotic caspase-3/7 to promote apoptosis. In this study, we demonstrate that in addition to its pyroptotic activity, GSDME augments caspase-3/7 activation and apoptotic cell death by targeting the mitochondria and releasing Cyt *c*. We further show that, like Bid, cleavage of GSDME by death receptor signaling bridges the extrinsic to the intrinsic apoptotic pathway. This novel function also appears to be conserved in GSDMD as its inflammasome-generated GSDMD-N domain is also capable of targeting the mitochondria and activating caspase-3/7 downstream of inflammasome activation. Lastly, using an in vivo mouse model of melanoma, we demonstrate that GSDME may possess a bona fide tumor suppressor activity as GSDME-deficient melanoma cells form and grow larger tumors than their wild-type (WT) counterparts.

## Results

### GSDME regulates caspase-3 activation

**Fig. 1:**
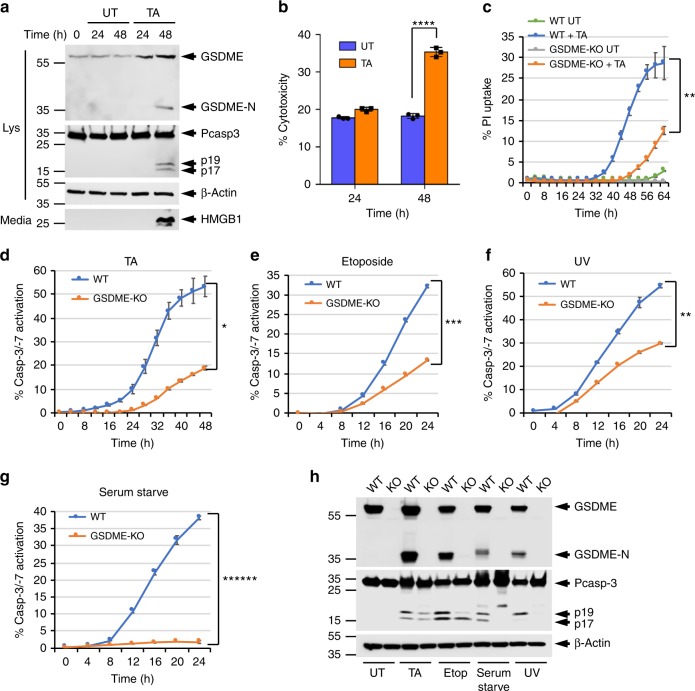
GSDME regulates caspase-3 activation and pyroptosis in CEM-C7 cells. **a** Immunoblots of GSDME, caspase-3, and β-actin in cell lysates (Lys), or high-mobility group box 1 (HMGB1) released in culture media (media) of CEM-C7 cells untreated (UT) or triamcinolone acetonide (TA) treated for the indicated times. **b** Cytotoxicity of TA as measured by lactate dehydrogenase (LDH) release in the culture supernatants of untreated (Untreated) or TA-treated (TA) CEM-C7 cells for the indicated times. **c**, **d** Propidium iodide (PI) uptake (**c**), and active caspase-3 staining (**d**–**g**) in wild-type (WT) and GSDME-knockout (KO) CEM-C7 cells treated with TA (**d**), etoposide (**e**), ultraviolet (UV) (**f**), or serum starvation (**g**) as measured on the IncuCyte over time. **h** Immunoblots of GSDME, caspase-3, and β-actin in combined cell lysates plus culture media  of WT and GSDME-KO (KO) CEM-C7 cells untreated (UT) or treated with TA, etoposide, serum starvation, or UV as indicated. Results are representative of at least three independent experiments performed in duplicate or triplicate. Error bars represent standard deviation (S.D.). Student’s *t*-test, *p < 0.05, **p < 0.005, ***p < 0.0005, ****p < 0.00005, ******p < 0.0000005

GSDME functions downstream of caspase-3 to switch apoptotic cell death to secondary necrotic/pyroptotic cell death^9,11^. To investigate whether GSDME plays additional roles in the apoptotic program, we studied its contribution to glucocorticoid-induced T-lymphoblastic leukemia cell killing^25,26^, because glucocorticoids have been shown to up-regulate GSDME messenger RNA in the human T-lymphoblastic leukemia cell line CEM-C7^27^. Treatment of CEM-C7 with the glucocorticoid triamcinolone acetonide (TA) induces upregulation and processing of GSDME (Fig. 1a, 1st panel), followed by induction of pyroptosis as measured by propidium iodide (PI) uptake (Fig. 1c), and release of HMGB1 (Fig. 1a, 4th panel) and lactate dehydrogenase (LDH; Fig. 1b) into the culture medium. Deletion of GSDME by CRISPR/Cas9 (clustered regularly interspaced short palindromic repeats/CRISPR-associated protein 9) gene editing (Supplementary Fig. 1a-c), compromised TA-induced pyroptosis in these cells (Fig. 1c). Interestingly, deletion of GSDME also considerably decreased the kinetics and magnitude of caspase-3 activation by TA treatment (Fig. 1d, h). These effects were not specific to TA treatment or to CEM-C7 cells but were also observed with other apoptotic stimuli in CEM-C7 cells (Fig. 1e–h, Supplementary Fig. 1d–f), and in other cell types such as immortalized bone marrow-derived macrophages (iBMDMs) (Supplementary Fig. 2), the murine melanoma cell line B16 (Supplementary Fig. 3), and the murine thymoma cell line EG7-Ova (Supplementary Fig. 4). Together, these results show that GSDME functions both downstream of caspase-3 to induce pyroptosis and upstream of caspase-3 to augment caspase-3 activation.

### GSDME activity is potentially regulated by phosphorylation

**Fig. 2:**
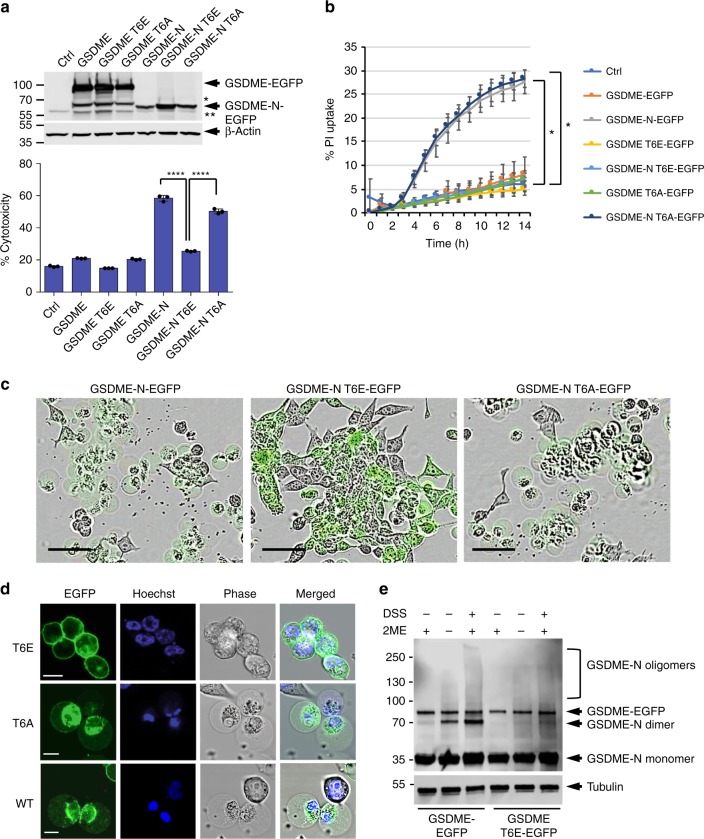
T6E mutation inhibits the pyroptotic and self-oligomerization activities of GSDME. **a**, **b** Cytotoxicity and pyroptotic activity of the indicated GSDME-N constructs as measured by lactate dehydrogenase (LDH) release (**a**) or propidium iodide (PI) uptake (**b**), respectively, in 293T cells transfected with empty vector (Ctrl) or the indicated GSDME. Expression of these constructs in cell lysates is shown in (**a**, top panel) as visualized by immunoblot analysis with anti-GSDME antibody. *Internal translation. **Non-specific band. **c** Representative IncuCyte images showing pyroptosis induction in cells expressing GSDME-N-EGFP (left) or GSDME-N-T6A-EGFP (right), but not in cells expressing GSDME-N-T6E-EGFP (middle). Scale bar, 50 µm. **d** Confocal images of GSDME-N-EGFP variants T6E (top panels), T6A (middle panels) and wild-type (WT; lower panels) transiently expressed in 293T cells. The perinuclear localization of WT or T6A GSDME-EGFP is largely due to the association of GSDME-N-EGFP with the mitochondria (see Fig. 4), which coalesce around the nucleus in pyroptotic cells. Scale bar, 10 µm. **e** Immunoblots showing WT or T6E GSDME-N after incubation with purified cell membranes and fractionation on sodium dodecyl sulfate (SDS) polyacrylamide gel in the presence or absence of β-mercaptoethanol (2-ME), or incubation with purified cell membranes followed by cross-linking with disuccinimidyl suberate (DSS) and fractionation on SDS–polyacrylamide gel in the presence of 2-ME. Results are representative of at least three independent experiments performed in duplicate or triplicate. Error bars represent S.D. Student’s *t*-test, *p < 0.05, ****p < 0.00005

To investigate whether posttranslational modifications may affect membrane targeting and killing activity of GSDME-N fragment, we searched the PhosphositePlus Resource (https://www.phosphosite.org) to identify all posttranslational phosphorylation sites in GSDME that were reported in high-throughput studies using proteomic mass spectrometry. This revealed that GSDME can be phosphorylated at multiple residues including T6, S69, S113, S114, T117, and S252. We next generated glutamate (phosphomimetic) or alanine mutants of these residues, and then measure their pyroptotic activity in HEK293T cells. Only the phosphomimetic T6E mutation significantly inhibited GSDME pyroptotic activity, while the alanine mutation had no effect (Fig. 2a–c, Supplementary Fig. 5a–e). As T6 has been shown to be phosphorylated^28^, and is a highly conserved residue in GSDME in all species examined (Supplementary Fig. 5f), it is likely that phosphorylation of this site plays an important physiological function.

We next used confocal microscopy and an in vitro oligomerization assay to examine the possibility that phosphorylation of T6 inhibits plasma membrane targeting and/or oligomerization of GSDME-N. Confocal microscopy revealed that GSDME-N T6E still localizes to the plasma membrane like WT and GSDME-N T6A but does not induce the necrotic swelling seen in cells expressing WT or GSDME-N T6A (Fig. 2d). However, the in vitro oligomerization assay showed that while WT GSDME-N forms dimers and high-molecular weight oligomers when incubated with heavy membrane fraction from HEK293T cells, GSDME-N T6E mutant fails to do so (Fig. 2e and Supplementary Fig. 6). These observations suggest that phosphorylation of T6 inhibits the pore-forming activity of GSDME-N by preventing its dimerization/oligomerization in membranes and this might be a critical regulatory mechanism to control the pyroptotic activity of GSDME and perhaps other gasdermin proteins. Indeed, T8 of GSDMA that corresponds to T6 in GSDME (supplementary Fig. 7a) is phosphorylated in response to activation of the Plk1 kinase^29^. Our results show that like GSDME-N, the pyroptotic activity of GSDMA-N is completely abolished by phosphomimetic mutation of T8 to glutamate (Supplementary Fig. 7c, d).

**Fig. 3:**
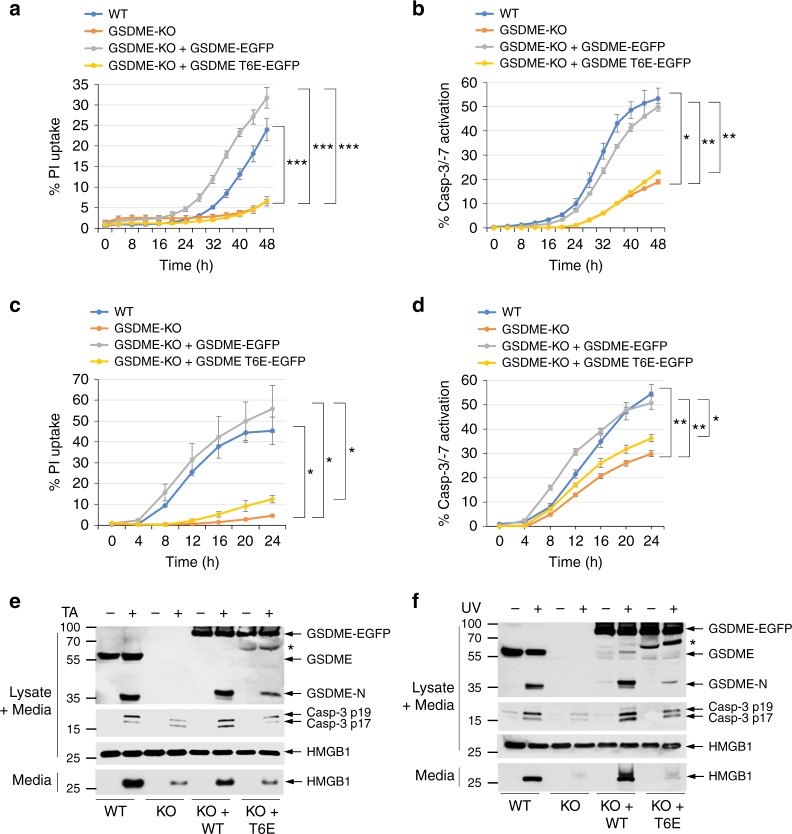
Pyroptosis and caspase-3 activation are compromised in GSDME-T6E-expressing CEM-C7 cells. **a**–**d** Propidium iodide (PI) uptake (**a**, **c**), and active caspase-3 staining (**b**, **d**) in CEM-C7 cells (wild-type (WT), GSDME-knockout (KO), or GSDME-KO reconstituted with WT GSDME-EGFP or GSDME-T6E-EGFP) treated with triamcinolone acetonide (TA) (**a**, **b**) or ultraviolet (UV) (**c**, **d**) as measured on the IncuCyte over time. **e**, **f** Immunoblots of GSDME, active caspase-3 p17/p19 and high-mobility group box 1 (HMGB1) in combined cell lysates plus media, or HMGB1 released in culture media (media) of WT (WT), GSDME-KO (KO), or GSDME-KO reconstituted with WT GSDME-EGFP (KO + WT) or GSDME-T6E-EGFP (KO+T6E) CEM-C7 cells treated with TA (**e**) or UV (**f**). *Indicates GSDME-EGFP degradation product. Results are representative of at least three independent experiments performed in duplicate or triplicate. Error bars represent S.D. Student’s *t*-test, *p < 0.05, **p < 0.005, ***p < 0.0005

We next investigated the effect of T6 phosphorylation on the kinetics of pyroptosis and caspase-3/7 activation in CEM-C7-knockout (KO) cells, stably reconstituted with WT or T6E mutant GSDME. As expected, cells expressing the T6E mutant GSDME exhibited diminished pyroptosis compared to WT cells or cells expressing WT GSDME in response to TA (Fig. 3a, e) or ultraviolet (UV; Fig. 3c, f) treatment as measured by PI uptake or release of HMGB1. The kinetics of PI uptake or HMGB1 release in T6E-expressing cells were comparable to those observed in GSDME-KO cells, confirming that T6 phosphorylation is critical for the pyroptotic activity of GSDME. Interestingly, cells expressing the T6E mutant also showed significant reduction in the kinetics and magnitude of caspase-3 activation in response to TA (Fig. 3b, e) or UV (Fig. 3d, f) treatment. This was also evident by reduced cleavage of the T6E GSDME mutant (Fig. 3e, f, 8th lane) compared to WT GSDME (Fig. 3e, f, 6th lane). These results indicate that the pore-forming activity of GSDME is not only required for pyroptosis but also required to enhance the kinetics and magnitude of caspase-3 activation in response to apoptotic stimuli.

### GSDME-N targets the mitochondria to release death proteins

**Fig. 4:**
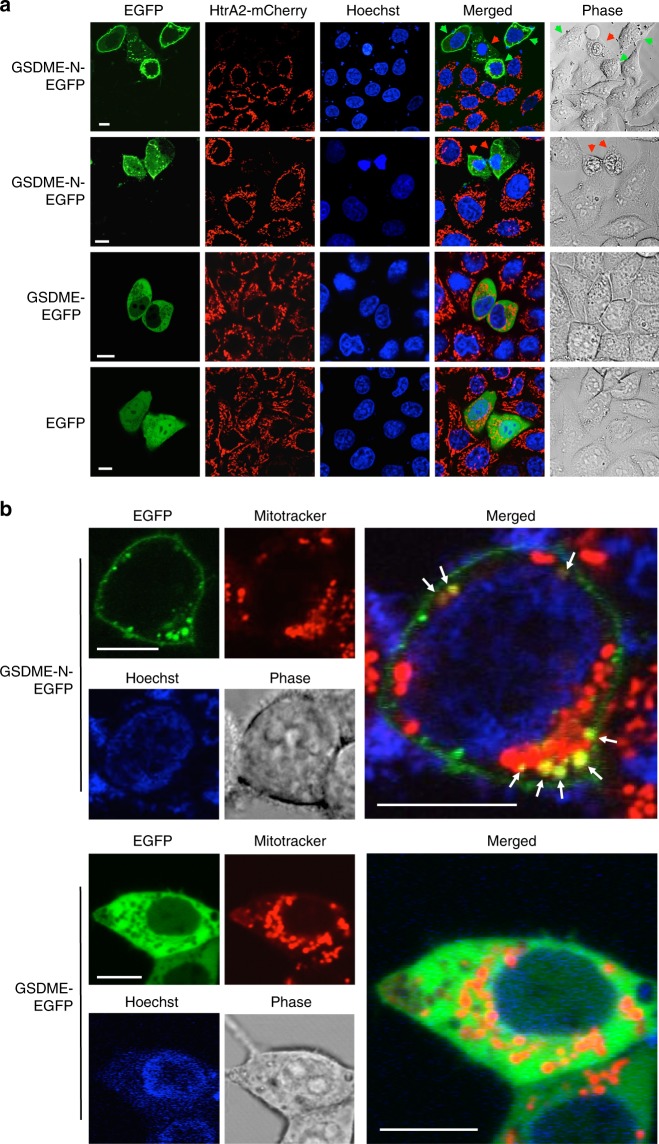
GSDME-N localizes to mitochondria and releases proapoptotic proteins. **a** Confocal live-cell imaging of GSDME-N-EGFP (1st and 2nd rows), GSDME-EGFP (3rd row), and EGFP (4th row) expressed in HeLa cells stably reconstituted with mCherry-tagged mitochondrial HtrA2. The green arrows indicate non-pyroptotic cells, and the red arrows indicate pyroptotic cells. **b** Confocal live-cell imaging of MitoTraker red-stained 293T cells after transfection with GSDME-N-EGFP (upper panels), or full-length GSDME-EGFP (lower panels). The green channels show the expression of GSDME-N-EGFP (upper left), or GSDME-EGFP (lower left). The red channels show mitochondrial staining with MitoTracker red. The merged channels are shown on the right. The arrows (upper right) indicate the co-localization of GSDME-N-EGFP with MitoTracker red. Results are representative of at least three independent experiments. Scale bar, 10 µm

To elucidate how GSDME potentiates caspase-3 activation during apoptosis, we investigated whether GSDME-N targets and permeabilizes the mitochondria to release apoptotic factors like Cyt *c* and HtrA2/Omi protease using confocal microscopy and biochemical analysis. Ectopic expression of GSDME-N-EGFP in HeLa cells that stably express mCherry-tagged HtrA2 caused a considerable reduction in the amount of mitochondrial HtrA2-mCherry compared to neighboring cells that do not express GSDME-N-EGFP or cells expressing full-length GSDME-EGFP or EGFP alone (Fig. 4a). Of particular interest, we observed release of mitochondrial HtrA2-mCherry in cells that did not yet show morphological features of pyroptosis (Fig. 4a, top panel, green arrows), suggesting that GSDME-N permeabilizes the outer mitochondrial membrane before plasma membrane rupture. In support of these observations, time-lapse live-cell imaging of HeLa-HtrA2-mCherry cells after transfection with GSDME-N-EGFP showed that expression of GSDME-N-EGFP induces early release of HtrA2-mCherry from the mitochondria into the cytosol before it ruptures the cell membrane (Supplementary Fig. 8, and Supplementary Movie 1).

Supporting the observation that GSDME-N targets the mitochondria, we observed overlap between GSDME-N-EGFP puncta and MitoTracker Red-stained mitochondria in GSDME-N-EGFP-expressing HEK293T cells (Fig. 4b, upper panels). Moreover, the MitoTracker Red signal was weaker in areas that overlapped with GSDME-N-EGFP, which is consistent with the ability of mitochondrial pore-forming proteins to decrease mitochondrial membrane potential. The full-length GSDME-EGFP showed cytosolic distribution and did not co-localize with MitoTracker red (Fig. 4b, lower panels), indicating that cleavage of GSDME is necessary for mitochondrial targeting. To provide further support that the GSDME-N fragment targets the mitochondria in cells undergoing apoptosis, we biochemically fractionated TNFα plus actinomycin D (TNFα/actD)-treated CEM-C7 cells. These experiments revealed that endogenous GSDME-N indeed localizes to the mitochondrial fraction during apoptosis (Supplementary Fig. 9).

**Fig. 5:**
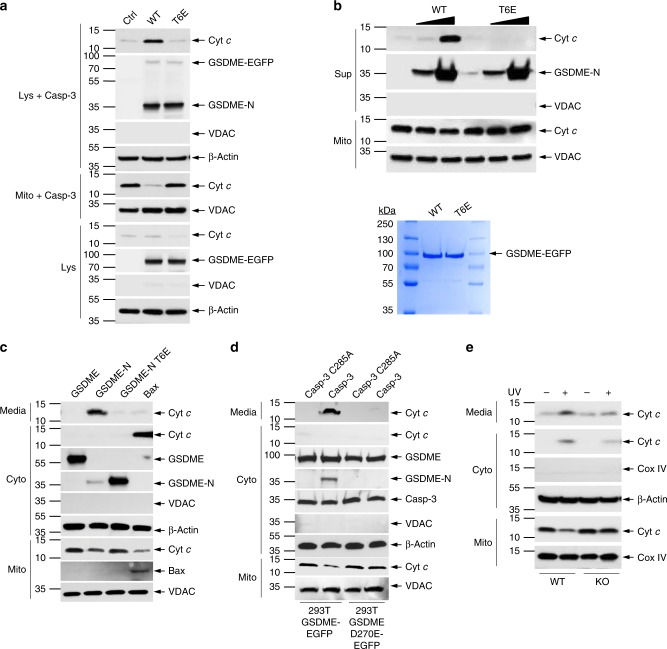
GSDME-N induces cytochrome *c* (Cyt *c*) release from the mitochondria. **a** Immunoblots of Cyt *c* (1st panel) released from purified mitochondria incubated with recombinant caspase-3, and S100 lysates from control (Ctrl) HEK293T cells or from HEK293T cells stably expressing wild-type (WT) GSDME-EGFP (WT) or GSDME-T6E-EGFP (T6E). (Lys+casp-3): S100 lysates after incubation with caspase-3 and mitochondria followed by removal of mitochondria by centrifugation. (Mito+casp-3): mitochondrial pellet obtained at the end of the reaction. (Lys): S100 lysates before incubation with caspase-3 or mitochondria. **b**, Upper Immunoblots of Cyt *c* (1st panel) released into the reaction supernatant (Sup) from purified mitochondria (Mito) incubated with or without caspase-3 and WT GSDME-N (WT) or inactive GSDME-N-T6E (T6E) mutant (2nd panel). **b**, Lower Coomassie-stained sodium dodecyl sulfate (SDS)-gel of the purified full-length WT GSDME-N-EGFP (WT) or inactive GSDME-N-T6E (T6E) used in this experiment. **c** Immunoblots of Cyt *c* (1st and 2nd panels) released into the culture media (media) or cytosol (Cyto) from 293T cells transfected with constructs encoding GSDME, GSDME-N, GSDME-N-T6E, or Bax. **d** Immunoblots of Cyt *c* (1st and 2nd panels) released into the culture media (media) or cytosol (Cyto) from stable 293T cells expressing WT GSDME (293T-GSDME) or the uncleavable GSDME-D270E (293-GSDME-D270E) transfected with constructs encoding constitutively active caspase-3 (Casp-3) or inactive caspase-3 mutant (Casp-3-C285A). **e** Immunoblots of Cyt *c* (1st and 2nd panels) released into the culture media (media) or cytosol (Cyto) from WT (WT) or GSDME-KO (KO) CEM-C7 cells treated with ultraviolet (UV) for 18 h. Voltage-dependent anion channel (VDAC) and cytochrome *c* oxidase IV (Cox IV) blots were used as loading controls for mitochondria. β-Actin blots were used as loading controls for cytosolic extracts or lysates. Quantitative analyses of Cyt *c* release in (**a**–**e**) are shown in Supplementary Fig. 16. Results are representative of at least three independent experiments

**Fig. 6:**
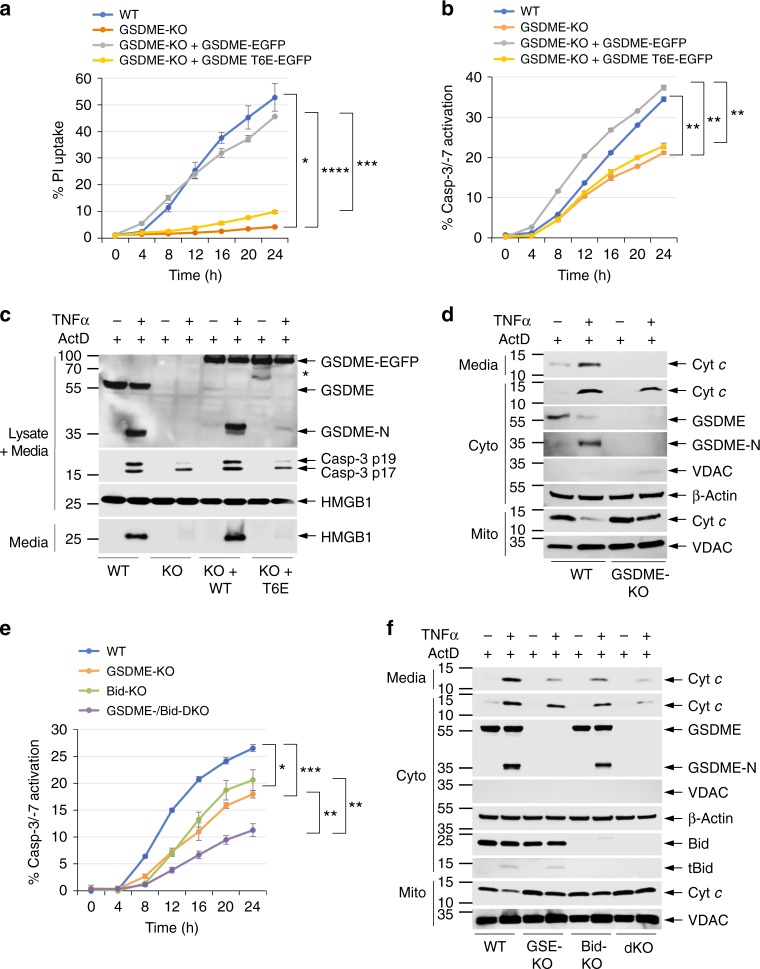
GSDME augments death receptor-induced pyroptosis and caspase-3 activation. **a**, **b** Propidium iodide (PI) uptake (**a**) and active caspase-3/7 staining (**b**) in CEM-C7 cells (wild-type (WT), GSDME-knockout (KO), or GSDME-KO reconstituted with WT GSDME-EGFP or GSDME-T6E-EGFP) treated with tumor necrosis factor α (TNFα) plus actinomycin D (ActD) as measured on the IncuCyte over time. **c** Immunoblots of GSDME, active caspase-3 p17/p19, and high-mobility group box 1 (HMGB1) in combined cell lysates plus media, or HMGB1 released in culture media (media) of WT (WT), GSDME-KO (KO), or GSDME-KO reconstituted with WT GSDME-EGFP (KO+WT) or GSDME-T6E-EGFP (KO+T6E) CEM-C7 cells treated with TNFα plus ActD as indicated. **d** Immunoblots of cytochrome *c* (Cyt *c*) (1st and 2nd panels) released into the culture media (media) or cytosol (Cyto) from WT (WT) or GSDME-KO (KO) CEM-C7 cells treated with TNFα plus ActD for 18 h. **e** Active caspase-3/7 staining in WT, GSDME-KO, Bid-KO, or GSDME/Bid-dKO CEM-C7 cells treated with TNFα plus ActD as measured on the IncuCyte over time. **f** Immunoblots of Cyt *c* (1st and 2nd panels) released into the culture media (media) or cytosol (Cyto) from WT, GSDME-KO (GSE-KO), Bid-KO, or GSDME/Bid-dKO (dKO) CEM-C7 cells treated with TNFα plus ActD. Quantitative analyses of Cyt *c* release in (**d**, **f**) are shown in Supplementary Fig. 17. Results are representative of at least three independent experiments performed in duplicate or triplicate. Error bars represent S.D. Student’s *t*-test, *p < 0.05, **p < 0.005, ***p < 0.0005, ****p < 0.00005

To provide direct evidence that GSDME-N can permeabilize the mitochondria to release death proteins, we incubated purified mitochondria with S100 extracts from HEK293T cells deficient in GSDME expression or stably expressing GSDME-EGFP or GSDME-T6E-EGFP in the presence of recombinant caspase-3. Upon cleavage of GSDME by caspase-3, the generated WT GSDME-N (Fig. 5a, 2nd lane), but not T6E GSDME-N mutant (Fig. 5a, 3rd lane), strongly induced release and depletion of Cyt *c* from the mitochondria (Fig. 5a, 2nd lane). Similar results were obtained when purified mitochondria were incubated with purified caspase-3-cleaved WT or T6E GSDME proteins (Fig. 5b). Furthermore, we looked at the release of Cyt *c* in HEK293T after transient expression of GSDME-N. As GSDME-N also forms pores in the plasma membrane, we saw extensive release of Cyt *c* into the culture media, accompanied by a depletion of mitochondrial Cyt *c* (Fig. 5c, 2nd lane). No Cyt *c* release was observed after expression of full-length GSDME or the GSDME-N T6E mutant (Fig. 5c, 1st and 3rd lanes). Cyt *c* release and depletion of mitochondrial Cyt *c* was also seen after expression of the known mitochondrial pore-forming protein Bax (Fig. 5c, 4th lane), but because Bax does not permeabilize the plasma membrane, Cyt *c* remained in the cytosol. We also observed that generation of the GSDME-N fragment by ectopic expression of active caspase-3^30^ in GSDME-EGFP-expressing HEK293T cells resulted in Cyt *c* release from the mitochondria into the culture media (Fig. 5d, 2nd lane). No Cyt *c* release was observed after expression of the catalytically inactive caspase-3 C285A mutant (Fig. 5d, 1st lane), or after expression of either active or inactive caspase-3 in cells that express the uncleavable GSDME-D270E-EGFP (Fig. 5d, 3rd and 4th lanes), indicating that generation of GSDME-N fragment by active caspase-3 is necessary for Cyt *c* release. Consistent with the above observations, we saw that UV or TNFα treatment induces more release and depletion of Cyt *c* from the mitochondria in WT cells compared to GSDME-KO CEM-C7 cells (Figs. 5e and 6d), indicating that physiological levels of GSDME-N can permeabilize the mitochondria.

Permeabilization of the mitochondrial membrane can also lead to increased mitochondrial reactive oxygen species (ROS) production^31^. Consistent with these observations, apoptotic stimuli induced more ROS production in WT and GSDME-EGFP-reconstituted GSDME-KO CEM-C7 cells compared with GSDME-KO and GSDME-T6E-EGFP-reconstituted GSDME-KO CEM-C7 cells (Supplementary Fig. 10). Collectively, our data indicate that in addition to its pyroptotic function, GSDME-N can also function as a mitochondrial pore-forming protein that contributes to mitochondrial permeabilization and Cyt *c* release during apoptosis.

### GSDME bridges extrinsic and intrinsic apoptotic pathways

Activation of caspase-8 in the extrinsic death receptor signaling complex can lead to both direct caspase-3 activation by caspase-8 (type I pathway) and indirect caspase-3 activation by a caspase-8-generated truncated Bid (tBid)-mitochondria-apoptosome amplification loop (type II pathway)^5,6,32–34^. GSDME-N, like tBid, might also function as a second amplification loop in the extrinsic pathway to potentiate caspase-3 activation. To test this hypothesis we stimulated the extrinsic pathway in GSDME-expressing and -deficient CEM-C7 cells with TNFα in the presence of actD, or TAK1 kinase inhibitors 5Z-7-Oxozeaenol or Takinib, which have been shown to potentiate TNFα-induced cell death^35,36^. Our results show that TNFα/actD, TNFα/5Z-7-Oxozeaenol, or TNFα/Takinib treatment induces more pyroptosis and caspase-3 activation in WT compared to GSDME-KO CEM-C7 cells (Fig. 6a–c and Supplementary Fig. 11b-e). Reconstitution of GSDME-KO cells with WT, but not T6E, GSDME restored pyroptosis and caspase-3 activation to levels comparable to those observed in WT cells (Fig. 6a–c). TNFα/actD stimulation also caused GSDME cleavage and more Cyt *c* release and depletion from the mitochondria in GSDME-expressing compared to GSDME-KO CEM-C7 cells (Fig. 6c, d), suggesting that the increased caspase-3 activation in WT GSDME-expressing compared to GSDME-KO cells is likely due to the ability of the GSDME-N fragment to target the mitochondria to induce Cyt *c* release.

Western blot analysis of the effect of TNFα/actD stimulation on GSDME and Bid in CEM-C7 cells showed no difference in the kinetics of GSDME and Bid cleavage (Supplementary Fig. 11a), suggesting that the GSDME and Bid amplification loops are activated simultaneously. To evaluate the contribution of these two loops to death receptor-induced cell death, we generated Bid-KO and GSDME/Bid-double knockout (DKO) CEM-C7 cell lines by CRISPR/Cas9 gene editing (Supplementary Fig. 12a, b). Deletion of Bid did not affect the pyroptotic function of GSDME as Bid-KO cells still retained the ability to take up PI after TNFα/actD treatment (Supplementary Fig. 12c). However, the kinetics of caspase-3 activation in the Bid-KO cells was significantly below those observed in WT cells, but comparable to those observed in GSDME-KO cells (Fig. 6e). Interestingly, combined deletion of Bid and GSDME further reduced the kinetics of caspase-3 activation (Fig. 6e), suggesting that they function independently of each other to amplify the mitochondrial pathway. Consistent with this, GSDME/Bid-DKO cells showed considerably less TNFα/actD-induced Cyt *c* release than that observed in either Bid-KO or GSDME-KO (Fig. 6f). Taken together, our results demonstrate that GSDME permeabilizes the mitochondria during death receptor signaling, thus bridging activation of the extrinsic and intrinsic apoptotic pathways.

### GSDME cleavage forms a self-amplifying feed-forward loop

**Fig. 7:**
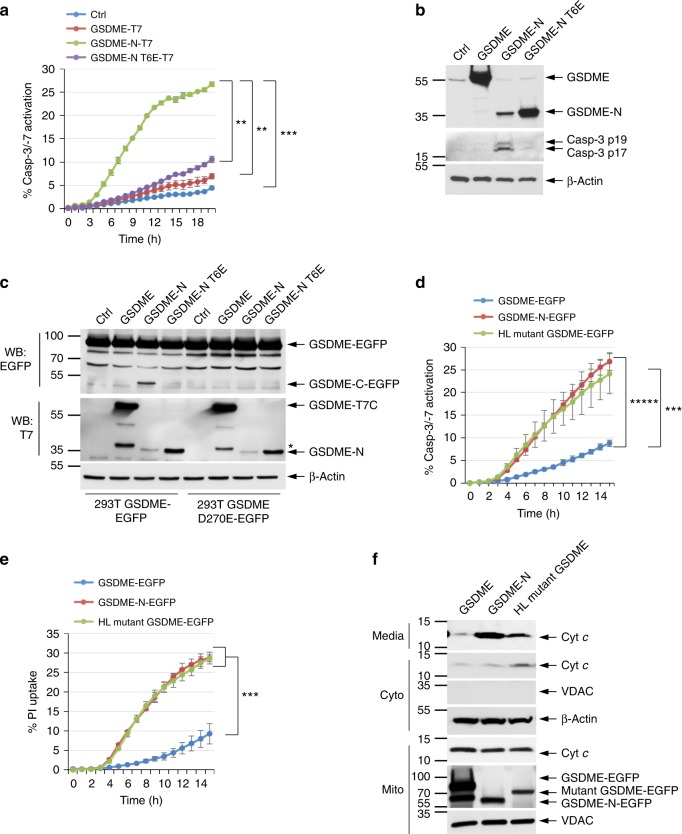
GSDME functions as a feed-forward amplifier of caspase-3 activation and pyroptosis. **a** Active caspase-3 in 293T cells transfected with empty vector (Ctrl) or the indicated GSDME constructs as measured on the IncuCyte over time. **b** Immunoblots of lysates from 293T cells transfected with the indicated constructs as in (**a**). **c** Immunoblots of lysates from stable 293T-GSDME-EGFP and 293T-GSDME-D270E-EGFP cells transfected with the indicated constructs encoding C-terminally T7-tagged GSDME proteins. The upper panel (probed with anti-EGFP) shows generation of the cleaved GSDME-C-EGFP domain in 293T-GSDME-EGFP, but not 293T-GSDME-EGFP-D270E cells in response to activation of caspase-3 by the transfected GSDME-N fragment. **d**, **e** Active caspase-3 staining (**d**) and propidium iodide (PI) uptake (**e**) in 293T cells transfected with the indicated GSDME constructs as measured on the IncuCyte over time. **f** Immunoblots of cytochrome *c* (Cyt *c*) (1st and 2nd panels) released into the culture media (media) or cytosol (Cyto) from 293T cells transfected with the indicated GSDME constructs. GSDME, full-length GSDME, GSDME-N, cleaved GSDME-N domain, Mutant GSDME, deafness-associated GSDME mutant. Quantitative analyses of Cyt *c* release in (**f**) are shown in Supplementary Fig. 18. Results are representative of at least three independent experiments performed in duplicate or triplicate. Error bars represent S.D. Student’s *t*-test, **p < 0.005, ***p < 0.0005, *****p < 0.000005

The ability of GSDME-N to permeabilize the mitochondria and enhance caspase-3 activation suggests that GSDME cleavage generates a self-amplifying, positive feed-forward loop. According to this model, the initially generated GSDME-N fragment releases Cyt *c* from the mitochondria, which in turn activates the Apaf-1 apoptosome, thereby causing further caspase-3 activation and cleavage of GSDME. Supporting this idea, GSDME-N fragment but not full-length or the inactive GSDME-N T6E mutant can activate caspase-3 when transiently expressed in 293T cells (Fig. 7a, b). All cells showing microscopic features of GSDME-N-mediated pyroptosis (cellular swelling and rounding) were also positive for caspase-3 staining (Supplementary Fig. 13a). Furthermore, WT GSDME-N fragment was only able to activate caspase-3 in WT mouse embryonic fibroblasts (MEFs) but not Apaf-1-KO MEFs (Supplementary Fig. 13b), indicating that GSDME-N-mediated caspase-3 activation is dependent on functional Apaf-1 apoptosome. Interestingly, when the GSDME-N was transiently expressed in HEK293T cells that stably express full-length WT GSDME-EGFP (293T-GSDME-EGFP cells), activation of caspase-3 by the GSDME-N fragment led to processing of GSDME-EGFP to generate additional GSDME-N fragments (Fig. 7c, 3rd lane). No additional GSDME-N fragments were generated when the inactive GSDME-N T6E mutant or full-length GSDME were transiently expressed in these cells or when GSDME-N was transiently expressed in cells expressing the uncleavable GSDME-D270E mutant (293T-GSDME-D270E-EGFP) (Fig. 7c, 7th lane), indicating that generation of additional GSDME fragments by the transiently expressed GSDME-N is dependent on caspase-3-mediated cleavage of full-length GSDME. Consistent with these results, induction of apoptosis in CEM-C7 cells that express the T6E mutant generates less GSDME-N fragment compared with cells that express WT GSDME (see Figs. 3e, f, and 6c, upper panels).

The above mechanism might explain how the human deafness-associated GSDME mutant induces cochlear cell death. Indeed, transient expression of deafness-associated GSDME mutant in 293T cells induced pyroptosis, caspase-3 activation, and Cyt *c* release from the mitochondria (Fig. 7d–f). Combined, our results demonstrate that GSDME-N can initiate a self-amplifying positive feed-forward loop via activation of the Apaf-1 apoptosome/caspase-3, which further cleaves full-length GSDME to enhance pyroptosis.

### Inflammasome-generated GSDMD-N activates caspase-3

**Fig. 8:**
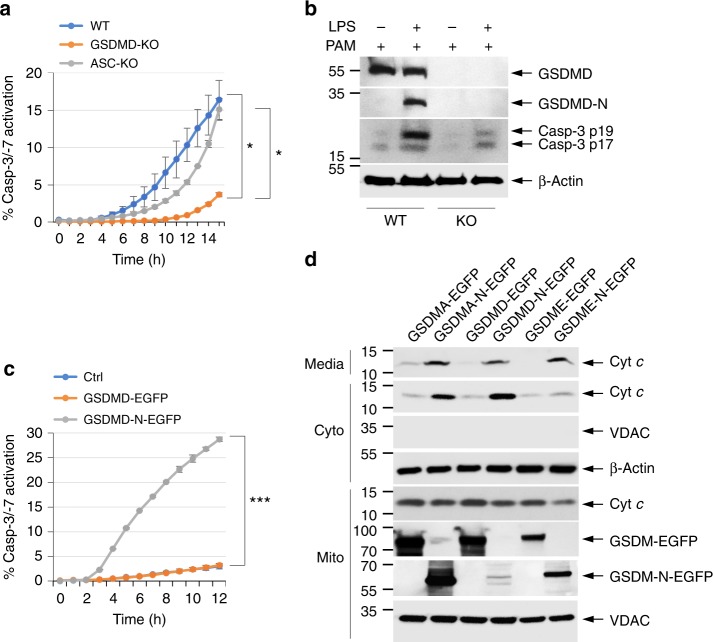
GSDMD functions downstream of the inflammasome to activate caspase-3. **a** Active caspase-3 staining in PamCSK4-primed wild-type (WT), GSDMD-knockout (KO), or ASC-KO immortalized bone marrow-derived macrophages (iBMDMs) transfected with lipopolysaccharide (LPS; caspase-11 activator) as measured on the IncuCyte over time. **b** Immunoblots of GSDMD, active caspase-3 p17/p19, and β-actin in cell lysates of PamCSK4-primed WT and GSDMD-KO (KO) iBMDMs untransfected (−) or transfected (+) with LPS as indicated. **c** Active caspase-3 in 293T cells transfected with empty vector (Ctrl) or the indicated GSDMD constructs as measured on the IncuCyte over time. **d** Immunoblots of cytochrome *c* (Cyt *c*; 1st and 2nd panels) released into the culture media (media) or cytosol (Cyto) from 293T cells transfected with the indicated GSDMA, GSDMD, or GSDME constructs. Quantitative analyses of Cyt *c* release in (**d**) are shown in Supplementary Fig. 19. Results are representative of at least three independent experiments performed in duplicate or triplicate. Error bars represent S.D. Student’s *t*-test, *p < 0.05, ***p < 0.0005

GSDMD is the mediator of pyroptosis downstream of canonical and non-canonical inflammasomes^7,8,10^. Interestingly, activation of the inflammasomes can also lead to activation of apoptotic caspase-3 and -7^37–40^. To investigate whether GSDMD cleavage downstream of non-canonical inflammasome activation contributes to caspase-3 activation, we measured the kinetics of caspase-3 activation after transfection of WT, GSDMD-KO, or ASC-KO iBMDMs with lipopolysaccharide (LPS)^41^. Remarkably, GSDMD deficiency significantly delayed and reduced the amount of LPS-induced caspase-3 activation (Fig. 8a, b). However, ASC deficiency had no notable effect, ruling out the involvement of ASC-dependent inflammasomes in the observed caspase-3 activation (Fig. 8a). GSDMD deficiency, but not ASC deficiency, also significantly reduced LPS-induced pyroptosis (PI uptake) and Cyt *c* release (Supplementary Fig. 14). These results indicate that GSDMD cleavage contributes to caspase-3 activation by the non-canonical inflammasome.

To show that GSDMD-N fragment can directly induce Cyt *c* release from the mitochondria and caspase-3 activation, we transiently expressed it in 293T cells and examined Cyt *c* release and caspase-3 activation. Similar to GSDME-N, expression of GSDMD-N, but not full-length GSDMD, resulted in Cyt *c* release and caspase-3 activation (Fig. 8c, d). Similar results were observed with GSDMA-N fragment (Fig. 8d and Supplementary Fig. 7b), indicating that mitochondrial targeting, Cyt *c* release, and caspase-3 activation is a general mechanism employed by the gasdermin proteins to potentiate apoptosis.

### GSDME may have a tumor suppressor activity

**Fig. 9:**
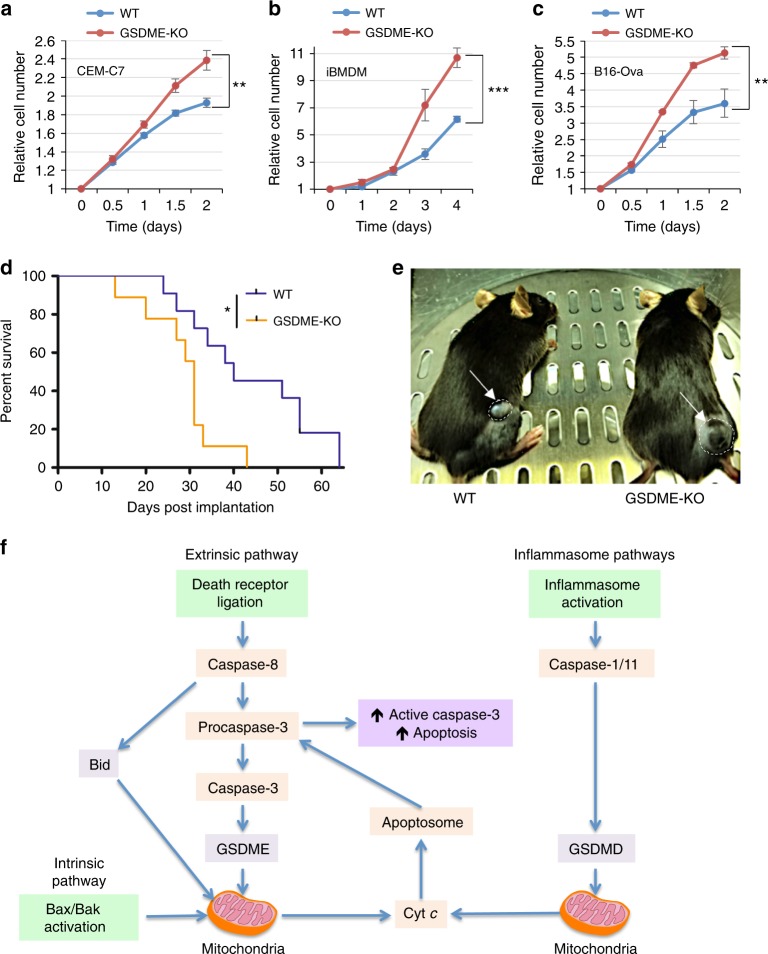
GSDME suppresses tumor cell growth in vitro and in vivo. Growth of wild-type (WT) and GSDME-KO CEM-C7 (**a**), immortalized bone marrow-derived macrophages (iBMDMs) (**b**), and B16-Ova (**c**) cells as measured on the IncuCyte over time. Growth curves are representative of at least three independent experiments performed in duplicate or triplicate. Error bars represent S.D. **d** Survival curve for mice inoculated with 3 × 10^5^ WT (*n* = 11 mice) or GSDME-KO (*n* = 9 mice) B16-Ova cells. Mice were killed once tumors reached volumes greater than 450 mm^3^. **e** A representative image of early tumor volume difference between WT and GSDME-KO tumors in mice. **f** A model representing how GSDMD and GSDME permeabilize the mitochondria to release cytochrome *c* (Cyt *c*) and augment caspase-3 activation and apoptosis. Cell growth curve significance was determined by Student’s *t*-test, **p < 0.005, ***p < 0.0005. Tumor survival curve significance was determined by log-rank test, *p < 0.05

Our results show that deletion of *GSDME* from CEM-C7, iBMDM, and B16-Ova cell lines increased their proliferative capacity (Fig. 9a–c). In addition, deletion of GSDME in B16-Ova cells enhanced their colony-forming ability after treatment with etoposide (Supplementary Fig. 15), suggesting that GSDME expression enhances overall cell death and reduces cell survival consistent with previous observations^18^. To further test if GSDME possesses a tumor suppressive activity in vivo, we utilized a mouse model of melanoma whereby WT C57BL/6J were subcutaneously injected with either WT or GSDME-KO B16-Ova melanoma cells and tumor growth was monitored over time. Consistent with the in vitro results, GSDME-KO tumors formed and grew to reach sacrificial threshold significantly faster than those expressing *GSDME* (Fig. 9d, e). These results suggest that *GSDME* possesses a tumor suppressive activity which might be related to its ability to induce pyroptosis and potentiate caspase-3 activation by activating the mitochondrial apoptotic pathway.

## Discussion

GSDME is believed to be a putative tumor suppressor^17,18,20^, but how it prevents tumorigenesis and why it is downregulated in many different cancer types has remained a mystery. In addition, although GSDME has recently been shown to form pores in the plasma membrane when cleaved by caspase-3^9,11^, this function occurs downstream of PCD pathways and does not explain its tumor suppressive activity.

In this study, we demonstrated that GSDME augments the intrinsic apoptotic pathway in cancer cells by forming pores in the mitochondria and releasing critical proapoptotic factors such as Cyt *c* and HtrA2. Confocal imaging of cells expressing GSDME-N-EGFP and mitochondrial HtrA2-mCherry show loss of HtrA2-mCherry from the mitochondria much earlier than the onset of plasma membrane ballooning, suggesting that GSDME-N may preferentially permeabilize the mitochondria before causing plasma membrane rupture. This observation might be explained by the fact that mitochondrial membranes contain cardiolipins, and gasdermin-N domain of several gasdermins including GSDME show strong preference for binding to cardiolipin and forming pores in mitochondria-like liposomes^11–14^. According to recent studies, gasdermin pores form inner diameters ranging from 10 to 18 nm depending on the lipid composition in which they insert themselves^12,42^. This should allow for the release of proapoptotic molecules such as Cyt *c* which has a diameter of ~3 nm^43^.

Our results demonstrated that GSDME augments apoptosis by stimuli that activate both the intrinsic and extrinsic apoptotic pathways (Fig. 9f). It amplifies intrinsic pathway signals by forming more pores in the mitochondria leading to enhanced Cyt *c* release and apoptosome activation. Similarly, it acts in parallel and in a manner analogous to Bid in the extrinsic pathway to augment Cyt *c* release and apoptosome activation. The ability of GSDME to augment apoptosis by targeting and permeabilizing the mitochondria suggests that it functions in a manner analogous to proapoptotic Bcl-2 family members^1,3^, which might explain its putative tumor suppressive activity. Indeed, our findings show that GSDME expression suppresses tumor cell growth in vitro and delays tumor formation and growth in vivo.

Besides triggering cell death, mitochondrial permeabilization has been reported to engage nuclear factor-κB anti-tumor signaling to cause tumor regression in vivo^44^. During transformation, developing tumor cells experience a variety of stresses such as hyperproliferation, excessive DNA damage, decreased survival factor signaling, and increased oncogene signaling that would normally result in activation of the apoptotic program and their own demise^45^. In order to survive these stresses, tumor cells downregulate or inactivate genes whose products permeabilize the mitochondria, such as the proapoptotic Bcl-2 proteins^45^. Interestingly, GSDME has also been reported to be downregulated in breast, gastric, and colorectal tumors^17,18,21,22^. This may represent an advantageous adaptation for tumor survival by reducing activation of intrinsic apoptosis and possibly anti-tumor signaling. It remains to be seen if mice deficient in GSDME are more prone to developing cancer.

Previous studies demonstrated that expression of the gain-of-function mutant GSDME in HEK293T cells induces apoptosis as evidenced by both flipping of phosphatidylserine to the outer leaflet and DNA fragmentation^46^. In addition, when expressed in *Saccharomyces cerevisiae*, this mutant colocalizes with mitochondria and causes elevated levels of ROS, oxidative stress, and cell death^47,48^. Moreover, other family members have also been suggested to activate the apoptotic program through the mitochondria. GSDMA, for example, has been shown to induce apoptosis in pit cells of the gastric epithelium during TGF-β signaling, and the N terminus of GSDMA3 has been shown to target the mitochondria via Hsp90 to induce oxidative stress and mitochondrial permeability transition^49,50^. Our findings are consistent with these reports and further provide more insights into the mechanism of how gasdermin proteins can cause apoptosis through permeabilizing the mitochondrial membrane.

Several reports have demonstrated the ability of the inflammasome-mediated pyroptotic pathway to activate the apoptotic pathway^37,38,40^. Our results provide a link between these two pathways by showing that GSDMD-N generated by inflammatory caspases targets the mitochondria to induce caspase-3 activation and apoptosis. Consistent with our findings, a previous study showed that upon activation of the non-canonical inflammasome in macrophages, GSDMD disrupts the mitochondrial membrane potential and leads to mitochondrial decay before rupture of the plasma membrane^51^. Interestingly, recent studies have also shown that GSDMD and GSDME are capable of directly lysing bacterial membranes^11–13^. The proapoptotic and bactericidal functions of these proteins could provide an enhanced innate immune response during infection by not only directly killing bacteria, but also killing the host cells harboring these pathogens. These mechanisms represent two important ways that cells can use to prevent the spread of bacteria during infection.

GSDME was discovered due to the finding that mutations in intron 7 lead to the development of sensorineural hearing loss due to exon 8 skipping which introduces a pre-mature stop codon that results in the translation of a C-terminally truncated protein^52,53^. As the gasdermin-C domain folds over and masks the pyroptotic gasdermin-N domain, the absence of the gasdermin-C domain in the GSDME hearing-loss mutant represents a gain-of-function mutation that unmasks its pore-forming activity independent of cleavage by caspase-3. Indeed, our data show that expression of this mutant is capable of releasing Cyt *c* from the mitochondria, activating caspase-3, and forming pores in the plasma membrane. GSDME is expressed in cochlear hair cells, which have a limited ability to regenerate themselves, and therefore cumulative mitochondrial damage caused by the unregulated activity of the mutant GSDME could result in a progressive decline in cochlear hair cell viability and function eventually leading to complete hearing loss^54^. Why mutant GSDME appears to only cause pathological damage to this specific tissue and not others could be a result of differences in GSDME expression, or tissue-specific differences in alternative splicing of exon 8 of GSDME.

Intriguingly, our data show that GSDME protein is upregulated in response to stimulation with the glucocorticoid (GC) TA in T-lymphoblastic leukemia CEM-C7 cells resulting in induction of pyroptosis and enhancement of caspase-3 activation. GCs are a class of steroid hormones that have been proposed to modulate several functions within the immune system ranging from positive and negative selection of thymocytes to thymocyte cell death induced by systemic concentrations of GCs reached during stress responses^55,56^. In addition, GCs are considered the cornerstone for the treatment of lymphoid cancers because of their ability to induce growth arrest and apoptosis in these cell types^57^. The mechanism of GC-induced apoptosis is not fully established, but it is now believed to be mediated in part by GC receptor-mediated transcriptional induction of proapoptotic Bcl-2 homology 3 (BH3)-only proteins^25^. Proapoptotic BH3-only proteins induce apoptosis by inhibiting anti-apoptotic Bcl-2 proteins such as Bcl-2 and Bcl-xL or activating proapoptotic Bcl-2 proteins such as Bax and Bak^58,59^. These interactions lead to oligomerization of Bax/Bak and their permeabilization of the mitochondrial membrane. Since GSDME is induced and cleaved during glucocorticoid-induced apoptosis, it is therefore likely that GSDME plays important physiological roles in determining the fate of developing thymocytes, the progression of lymphoproliferative disorders, or immune responses to stress. Thus, expression of GSDME in lymphoid malignancies might be an important determining factor in their response to glucocorticoid treatment.

A number of high-throughput mass spectrometric studies identified critical residues in gasdermins that are phosphorylated by cellular kinases, suggesting that these posttranslational modifications may regulate gasdermin protein activities. Two of these residues, T6 of GSDME and T8 of GSDMA, are highly conserved in all  species. Indeed, our studies revealed that phosphorylation of T6 in GSDME or T8 in GSDMA appears to be an important regulatory mechanism to inhibit their mitochondria- and plasma membrane-pore-forming functions. The kinases responsible for direct phosphorylation of these residues have not yet been characterized, but one study has shown that activation of the serine/threonine kinase PLK1 (polo-like kinase 1) induces phosphorylation of many cellular proteins including GSDMA^29^. PLK1 is a proto-oncogene and an important regulator of the G2/M transition during cell cycle progression. Pharmacological inhibition or small interfering RNA-mediated depletion of PLK1 in cancer cells results in activation of the apoptotic pathway^60,61^. If PLK1 can directly or indirectly phosphorylate gasdermin family members, then this may inhibit their pore-forming activity during mitosis or during oncogenesis to prevent cell death. Recently, a 3.8 Å cryo-electron microscopy structure of the GSDMA3 pore was solved by Ruan et al.^42^, demonstrating that a 27-fold symmetry pore is formed in lipid bilayers. Interestingly, they observed that helix α1 of GSDMA3 monomers juxtapose end-on through hydrogen bonding and hydrophobic interactions in the oligomerized pore conformation. The structure also revealed that α1 is important for oligomerization. Specifically, α1 makes contact at oligomerization interface II with adjacent GSDMA3-N monomers to stabilize pore formation. As our GSDME-T6E mutant still retains its ability to localize to the plasma membrane (Fig. 2d), it is likely that phosphorylation of GSDME T6 or the equivalent GSDMA3 T8 residue introduces a negative charge that pushes α1 away from the beta-barrel domain and disrupts the oligomerization interface II. Indeed, our results demonstrated that phosphomimetic mutation of T6E of GSDME inhibits its dimerization/oligomerization and pore-forming and apoptotic activities

In conclusion, our work provides a conceptual advance in understanding the function of GSDME in the apoptotic program and tumor suppression. We have demonstrated that GSDME is an important and novel mitochondrial pore-forming protein that augments activation of the apoptotic pathway by releasing Cyt *c* from the mitochondria and activating the intrinsic apoptotic pathway. This function appears to be shared by other family members such as GSDMD, which too can activate the intrinsic pathway downstream of inflammasome activation. Future studies should focus on how GSDME can be exploited to predict the responsiveness of cancers to different therapeutics (i.e., lymphoid cancers to glucocorticoid treatment) and to design novel drugs that target this pathway.

## Methods

### Antibodies and reagents

T7˙Tag monoclonal antibody horseradish peroxidase (HRP)-conjugate (Cat. No. 69048) was from Novagen. Anti-DFNA5/GSDME (Cat. No. ab215191), anti-GSDMD (Cat. No. ab209845), anti-GFP-HRP (Cat. No. ab6663), anti-HMGB1 (Cat. No. ab18256), and anti-VDAC1 (Cat. No. ab154856) were from Abcam. Caspase-3 (Cat. No. 9662) and Cox IV (Cat. No. 4844) were from Cell Signaling Technologies. Anti-Bax (Cat. No. sc-930) and anti-GSDMA (Cat. No. sc-376318) were from Santa Cruz. Anti-cytochrome *c* (Cat. No. 556433) was from BD Biosciences. Anti-tubulin (Cat. No. CP06) was from Oncogene. All antibodies for western blot analyses were used at 1:1000 dilution except for anti-β-actin which was used at 1:10,000. Monoclonal anti-β-actin (Cat. No. A5441), propidium iodide (Cat. No. P-4170), triamcinolone acetonide (Cat. No. T-6501), 5Z-7-Oxozeaenol (Cat. No. O9890), and actinomycin D were obtained from Sigma Aldrich. TNFα (Cat. No. 210-TA-020) and anti-caspase-8 antibody (Cat. No. AF1650) were obtained from R&D Systems (Cat. No. 210-TA-020). Etoposide (Cat. No. A1971) was obtained from ApexBio. CytoTox96 LDH release kit (Cat No. G1780) was from Promega. In-Fusion HD Cloning Plus (Cat. No. 638910) was obtained from Takara Clontech. IncuCyte® Caspase-3/7 Green and Red Apoptosis Assay Reagents (Cat. Nos. 4440 and 4704) were obtained from Essen BioScience. MitoTracker Red CMXRos (Cat. No. M7512) and Hoechst (Cat. No. I34406) were obtained from Invitrogen. DSS (Cat. No. 21555) and CellROX^TM^ Deep Red Reagent (Cat. No. C10422) were obtained from Thermo Scientific. Ultrapure LPS and Pam3CSK4 were obtained from InvivoGen.

### Cell culture and treatments

CEM-C7 (a derivative of CCRF-CEM, ATCC® CCL-119™)^62^, B16-Ova (a derivative of B16F0, ATCC® CRL-6322™)^63^, and E.G7-Ova (ATCC® CRL-2113™) cells were cultured in RMPI 1640 medium (Gibco) supplemented with 10% fetal bovine serum (FBS), 10 mM HEPES pH 7.0 (Invitrogen), 100 U/mL penicillin and streptomycin, 1 mM sodium pyruvate (Cellgro), 2 mM l-glutamine, and 0.1% 2-mercaptoethanol (Gibco). Bone marrow-derived cells were harvested from the femurs of WT (C57BL/6) and knockout mice and differentiated into BMDMs by culturing in Dulbecco's modified Eagle's medium (DMEM; Gibco) supplemented with 10% FBS, 10 mM HEPES pH 7.0 (Invitrogen), 100 U/mL penicillin and streptomycin (complete DMEM), and 20% L929 supernatants in 10 cm dishes at 37 °C with 5% CO_2_ for 5–6 days. Immortalized BMDMs were generated by transformation of primary BMDMs with J2-CRE retrovirus^64^. The 293T cells were cultured in DMEM/F12 (Gibco) supplemented with 10% FBS, 10 mM HEPES pH 7.0 (Invitrogen), and 100 U/mL penicillin and streptomycin. HeLa HtrA2-mCherry cells were cultured in complete DMEM and WT and Apaf-1-KO MEFs were cultured in complete DMEM supplemented with 2 mM l-glutamine. For caspase-3/7 activation and PI uptake kinetic experiments, 1 × 10^5^ iBMDMs or B16-Ova cells were seeded in 96-well plates overnight or 1 × 10^5^ CEM-C7 or E.G7-Ova were seeded the same day. On the day of treatment, 1:1000 dilution of caspase-3/7 apoptosis reagent, 5 µM CellROX^TM^ Deep Red Reagent, or 1.5 µM propidium iodide with or without 1 µg/mL actinomycin D or 125 nM 5Z-7-Oxozeaenol, and 1 ng/mL TNFα, 10 µg/mL triamcinolone acetonide, or 150 µM etoposide was added to cells or cells were exposed to 22 mJ/cm^2^ UV irradiation in a UV Stratalinker® 1800. Plates were placed in the IncuCyte® S3 Live Cell Analysis System and imaged at 20× magnification. Signal percentage was calculated with the formula (percent red confluence/percent phase confluence) × 100. Unless otherwise indicated, background signal from untreated samples was subtracted from treated sample signal.

For transfection experiments, 293T cells were seeded overnight in 6-well plates at a density of 6 × 10^5^ cells per well. The next day, cells were transfected with 500 ng of plasmid DNA using Lipofectamine 2000 (7 μL/mL) as per the manufacturer’s protocol (Invitrogen) in 1 mL of Opti-MEM per well. For Incucyte imaging, 1:1000 dilution of caspase-3/7 apoptosis reagent or 1.5 µM propidium iodide was added to cells after transfection and the plates were placed in the IncuCyte® S3 Live Cell Analysis System and imaged at 20× magnification. The culture supernatants and cells were collected 18–24 h later for western blot analysis.

### GSDME oligomerization

Stable 293T, 293T GSDME-EGFP, or 293T GSDME-T6E-EGFP cell pellets were resuspended in 2.5× volume of Buffer A (250 mM sucrose, 20 mM HEPES, 10 mM KCl, 1.5 mM MgCl_2_, 1 mM EDTA, 1 mM EGTA, 1 mM dithiothreitol, 0.1 mM phenylmethylsulfonyl fluoride, pH 7.5) and left on ice for 15 min. Cells were lysed by passing through a syringe (30×) using a 26 G needle. The lysates were centrifuged at 800 × *g* for 10 min at 4 °C in a refrigerated Eppendorf 5417 R centrifuge. The 293T supernatant was collected and further centrifuged at 7000 × *g* for 10 min to pellet heavy membranes. The resulting pellet was washed with Buffer A, divided into 2 tubes, and spun again. GSDME-EGFP and GSDME-T6E-EGFP supernatants were further centrifuged at 100,000 × g for 10 min at 4 °C and the resulting supernatants were designated S100 lysates. To generate GSDME cleavage, 100 µL reactions were set up with recombinant caspase-3 and incubated at 37 °C for 1.5 h. To facilitate oligomerization, reactions were then added to the heavy membrane pellet from 293Ts and incubated at 37 °C for an additional 30 min. Reactions were centrifuged at 7000 × *g* for 10 min. Pellets were washed in Buffer A, centrifuged again, and resuspended in 100 µL Buffer A. Then, 20 µL of the resuspended material was incubated with 3 mg/mL disuccinimidyl suberate (DSS) for 30 min at room temperature, and 20 µL of 2× Laemmli buffer with 2-mercaptoethanol (2-ME) was added to quench the reaction. The 2× Laemmli buffer containing or not containing 2-ME was also added to two separate 20 µL suspensions, and these reactions were analyzed by sodium dodecyl sulfate–polyacrylamide gel electrophoresis (SDS–PAGE).

### Generation of CRISPR/Cas9 knockout cell lines

The CRISPR design tools at MIT (http://crispr.mit.edu) or Benchling (https://benchling.com) were used to identify candidate single-guide RNA (sgRNA) sequences. Sequences targeting human *GSDME* exon 1 (5’-AGTACCAGTTTTTATCCCTC-3’ and 5’-TCACCCTTGGCGATGTACTC-3’) or exon 3 (5’-CGTAGAGAGCCAGTCTTCAT-3’), murine *GSDME* exon 3 (5’-GTGTGAGAACCATAAGAGCG-3’ and 5’-GGGCTATTGGGACAGTCGTG-3’), and human *Bid* exon 2 (5’-CAACAACGGTTCCAGCCTCA-3’ and 5’-CAGGGATGAGTGCATCACAA-3’) were cloned into lentiCRISPRv2GFP vector (Addgene) containing Cas9 fused to EGFP. Next, 5 µg lentiCRISPRv2GFP sgRNA plasmids were cotransfected with 3.75 µg psPAX2 and 2.5 µg VSVg plasmids (Addgene) using 25 µL Lipofectamine 2000 to 2 × 10^6^ 293Ts seeded the day before. At 72 h after transfection, media were collected and concentrated overnight using Lenti-X Concentrator (Takara Cat. No. 631232) as per the manufacturer’s protocol. Infected 1 × 10^6^ CEM-C7 or 2.5 × 10^4^ B16-Ova cells/mL were plated in a 12-well plate with concentrated lentivirus. Cells were then enriched for Cas9-EGFP expression by flow cytometry and single cells were then isolated and screened by western blot analysis for protein expression. Sequencing was performed on PCR-amplified genomic DNA encompassing the sgRNA targeting sequences.

### Generation of constructs and stable cell lines

GSDMA, GSDMD, and GSDME constructs were made in pcDNA3, pEGFPN1, or pMSCVpuro EGFPN1 vectors using the In-Fusion PCR cloning kit with appropriate PCR primers and full-length human GSDMA, human or mouse GSDME, or mouse GSDMD complementary DNAs (cDNAs). The human GSDMA cDNA was obtained from TransOMIC (MGC premier cDNA clone for GSDMA–BC109197, Cat. No. TCH1003), the human GSDME cDNA was obtained from TransOMIC (MGC premier cDNA clone for GSDME–BC125065, Cat. No. TCH1003), the mouse GSDME cDNA was obtained from TransOMIC (MGC premier cDNA clone for GSDME–BC132303, Cat. No. TCM1003), and the mouse GSDMD cDNA was obtained from Dharmacon (MGC Mouse GSDMD cDNA clone ID 4194837, Cat. No. MMM1013–202765078). Stable GSDME-EGFP and GSDME-D270E-EGFP cell lines were generated by transfecting pEGFPN1-GSDME or pEGFPN1-GSDME-D270E plasmids followed by multiple cell sorting over a period of 1 month by flow cytometry.

### LDH release assay

Pyroptosis was quantitated by assaying the activity of LDH released into cell culture supernatants after various treatments and transfections using the CytoTox96 LDH release kit (Promega) according to the manufacturer’s protocol. The LDH activity in the culture supernatant was expressed as a percentage of total LDH in the cell lysate.

### Immunoblot analysis

CEM-C7, E.G7-Ova, iBMDM, and 293T cells were lysed in buffer containing 50 mM Tris, pH 7.5, 150 mM NaCl, 1 mM EDTA, 0.1% NP-40, and protease inhibitors and clarified by spinning at 14,000 RPM for 10 min at 4 °C in an Eppendorf tabletop 5417R refrigerated microcentrifuge. B16-Ova cells were lysed by adding 2× Laemmli buffer. Cell lysates were fractionated by SDS–PAGE and then transferred to polyvinylidene difluoride (PVDF) membranes (Bio-Rad).

To examine HMGB1 release, cell culture supernatants were precipitated by methanol/chloroform method^64,65^. Briefly, culture media were spun at 2000 RPM for 5 min to pellet cells and cell debris. Supernatants were transferred to a fresh tube and proteins were precipitated by the addition of an equal volume of methanol and 0.25 volumes of chloroform to each sample followed by centrifugation at 12,000 RPM for 10 min. The upper phase was discarded and the same volume of methanol was again added to the interphase of each sample followed by centrifugation for 5 min at 14,000 RPM. The resulting protein pellets were dried at room temperature, resuspended in 2× Laemmli buffer, and boiled for 10 min at 99 °C until dissolved. The resuspended proteins were fractionated on 12% SDS–PAGE followed by electroblotting onto PVDF membranes. Blots were probed with appropriate antibodies.

To examine total cleaved caspase-3 after treatment, cells were first lysed in their culture media by adding Triton X-100 to each well to a final concentration of 0.8%. Proteins were precipitated by methanol/chloroform extraction and prepared for western blot analysis as described above. Full size images are presented in Supplementary Figs. 20–33.

### Confocal microscopy

4 × 10^5^ 293T or 2 × 10^5^ HeLa HtrA2-mCherry cells were seeded on 35 mm cover glass-bottom culture dishes and allowed to attach for 24 h. Cells were transfected with constructs for different GSDME mutants for 18–24 h using Lipofectamine 2000 and then stained with Hoechst 33342 and/or MitoTracker Red CMXRos for 30 min. Cells were observed using a Nikon A1R resonant scanning confocal microscope (Bioimaging Shared Resource of the Kimmel Cancer Center (NCI 5 P30 CA-56036)). Live-cell time course was performed by taking images every 10 min for 7 h.

### Cytochrome *c* release assay

After treatment or transfection, cells were collected and washed in phosphate-buffered saline (PBS). Pellets were then resuspended in 5× volume of cytosolic extraction buffer (250 mM sucrose, 70 mM KCl, 137 mM NaCl, 4.3 mM Na_2_HPO_4_, 1.4 mM KH_2_PO_4_, 300 µg/mL digitonin, and protease inhibitors) and incubated on ice for 5 min. Samples were centrifuged at 1000 × *g* at 4 °C for 5 min. The supernatant was collected and run as the cytosolic fraction. Pellets were then resuspended in 5× volume of mitochondrial lysis buffer (50 mM Tris, pH 7.4, 150 mM NaCl, 2 mM EDTA, 2 mM EGTA, 0.2% Triton X-100, 0.3% NP-40, and protease inhibitors) and incubated on ice for 10 min. Samples were then spun at 10,000 × *g* at 4 °C for 10 min. The supernatant was collected and run as the mitochondrial fraction. The culture media were also collected, precipitated by methanol/chloroform as described above, and run as the media fraction.

### GSDME-EGFP purification and mitochondrial permeabilization assays

GSDME-EGFP or GSDME-T6E-EGFP were purified on GFP-Trap Agarose beads (Chromotek Cat. No. gta-20) according to the manufacturer’s protocol. Briefly, stable 293T GSDME-EGFP or GSDME-T6E-EGFP cells were lysed in 10 mM Tris, pH 7.5, 150 mM NaCl, 0.5 mM EDTA, 0.5% NP-40 supplemented with protease inhibitors and spun at 20,000 × *g* for 10 min. Supernatant was collected and diluted with 10 mM Tris, pH 7.5, 150 mM NaCl, and 0.5 mM EDTA. Dilutions were rotated for 1 h at 4 °C with 50 µL GFP-Trap agarose beads followed by 3 washes with dilution buffer. To elute GSDME-EGFP, beads were mixed with 50 µL 0.2 M glycine, pH 2.5 for 30 s. After spinning, the supernatant was collected and neutralized with the addition of 5 µL 1 M Tris, pH 10.4.

Mitochondria were purified by resuspending 293T cells in 2.5× volume of buffer A and incubating on ice for 15 min. The cells were lysed by syringing (30×) with a 26 G needle. The lysates were centrifuged at 800 × *g* for 10 min at 4 °C in a refrigerated Eppendorf 5417 R centrifuge. The 293T supernatant was collected and further centrifuged at 7000 × *g* for 10 min to pellet mitochondria. Pellet was resuspended in 2.5× volume of buffer A. The 20 µL reactions were set up with 1 or 5 µL purified GSDME-EGFP, 5 µL mitochondria, and recombinant caspase-3 and incubated at 37 °C for 1 h. Reactions were spun at 7000 × *g* for 10 min. The supernatant was collected, and the pellet was resuspended in 20 µL buffer A. Then, 2× Laemmeli buffer was added to each fraction and analyzed via western blot.

S100 mitochondria permeabilization experiments were performed by resuspending stable 293T GSDME-EGFP or GSDME-T6E-EGFP cells in 2.5× volume buffer A and incubating on ice for 15 min. The cells were lysed by syringing (30×) with a 26 G needle. The lysates were centrifuged at 800 × *g* for 10 min. Supernatants were collected and spun at 100,000 × *g* for 10 min. Then, 20 µL reactions were set up by adding 5 µL mitochondria purified as described above to 14 µL S100 lysate and recombinant caspase-3. Reactions were incubated at 37 °C for 1 h and spun at 7000 × *g* for 10 min. Fractions were then analyzed as above.

### LPS transfection experiment

2 × 10^6^ of WT, ASC-KO, or GSDMD-KO iBMDMs were seeded in a 6-well plate and allowed to attach overnight. Cells were pretreated with 1 µg/mL Pam3CSK4 for 5 h. Media were then replaced with fresh media containing 1:1000 dilution of caspase-3/7 apoptosis reagent followed by transfection of 2 µg LPS using 7 µL/mL of Lipofectamine 2000 according to the manufacturer’s protocol. Cells were then imaged every hour on the IncuCyte® S3 Live Cell Analysis System at 20× magnification.

### Cell fractionation for membrane localization

2 × 10^7^ CEMs were treated with TNFα/actD for ~18 h. Pellets were then washed in PBS, resuspended in 5 volumes of Buffer A, and left on ice for 15 min before lysing by syringing through a 26 G needle (30×). The cell lysates were centrifuged at 800 × *g* for 10 min at 4 °C in a refrigerated eppendorf 5417R centrifuge. The resulting supernatants were then transferred and centrifuged at 7000 × *g* for 10 min. The resulting pellets containing heavy membranes (P7) were washed and then resuspended in the same volumes of buffer A. The supernatants (S7) were further centrifuged at 20,000 × *g* for 10 min and the resulting pellets containing light membranes (P20) were washed and then resuspended in the same volumes of buffer A. The supernatants (S20) were further centrifuged at 100,000 × *g* for 10 min. The resulting supernatants (S100) and pellets (P100) were collected and the pellets were then washed and resuspended in the same volumes of buffer A. All fractions were then analyzed by SDS–PAGE followed by immunoblotting with GSDME antibody.

For sucrose step density gradient purification of mitochondria, cells were treated as above. The washed p7 pellet was then resuspended in 100 µL of Buffer A and added to the top of a sucrose step density gradient consisting of 5 mL of a 1 M sucrose solution layered on top of a 1 mL of 1.5 M sucrose solution. The gradient was then centrifuged at 22,000 RPM for 20 min at 4 °C in an SW 41 Ti swinging bucket rotor. The top of the 1 M sucrose layer containing plasma membrane fraction and the interface between the 1 M and 1.5 M sucrose layers containing the mitochondria fraction were collected, diluted to 0.25 M sucrose with a 5 mM Tris-HCl and 1 mM EDTA solution. These diluted fractions were then centrifuged at 17,000 × *g* for 15 min at 4 °C. The resultant pellets were resuspended in equal volumes of buffer A and analyzed by SDS–PAGE.

### Colony formation assay

Colony formation was determined by Clonogenic assay^66^. The 500 WT or GSDME-KO B16-Ova cells were seeded in a 6-well plate and allowed to attach at 37 °C with 5% CO_2_ for 4 h. Cells were then treated with 500 nM etoposide for 4 h. Wells were washed, replaced with complete media, and cells were grown for 7 days. Wells were imaged on the IncuCyte and colonies containing >50 cells were counted.

### Melanoma grafts

3 × 10^5^ WT or GSDME-KO B16 melanoma cells^67^ were injected subcutaneously into the lower flanks of C57BL/6 mice. Tumors volumes were measured every 2–3 days using the formula *V* = 0.52 (length × width^2^). Animals were killed when tumors reached volumes greater than 450 mm^3^. Survival curves were generated from mice that formed tumors. All animal studies were performed at Thomas Jefferson University and approved by Institutional Animal Care and Use Committee, and comply with all relevant ethical regulations for animal testing and research.

### Statistics

Statistical analyses were made with Student’s *t*-test and log-rank test.

## Supplementary information

Supplementary information

Supplementary Movie 1

Source Data

## Data Availability

The data that support the findings of this study are available from the corresponding author on reasonable request. Source data for graphs presented in figures are provided in the Source Data file.
