# Supplementary material for: Gasdermin pores permeabilize mitochondria to augment caspase-3 activation during apoptosis and inflammasome activation
Source: Nat Commun. 2019 Apr 11;10:1689. doi: 10.1038/s41467-019-09397-2 (PMC6459836; doi:10.1038/s41467-019-09397-2)
Supplement: Supplementary file 1 — Supplementary information [file 41467_2019_9397_MOESM1_ESM.pdf]

Supplementary Information

Gasdermin pores permeabilize mitochondria to augment caspase-3 activation during apoptosis and inflammasome activation

Corey Rogers[*], Dan A. Erkes[†], Alexandria Nardone[*], Andrew E. Aplin[†], Teresa Fernandes-Alnemri[*1], & Emad S. Alnemri[*1]

[*]Department of Biochemistry and Molecular Biology and [†]Department of Cancer Biology, Sidney Kimmel Cancer Center, Thomas Jefferson University, Philadelphia, PA 19107, USA

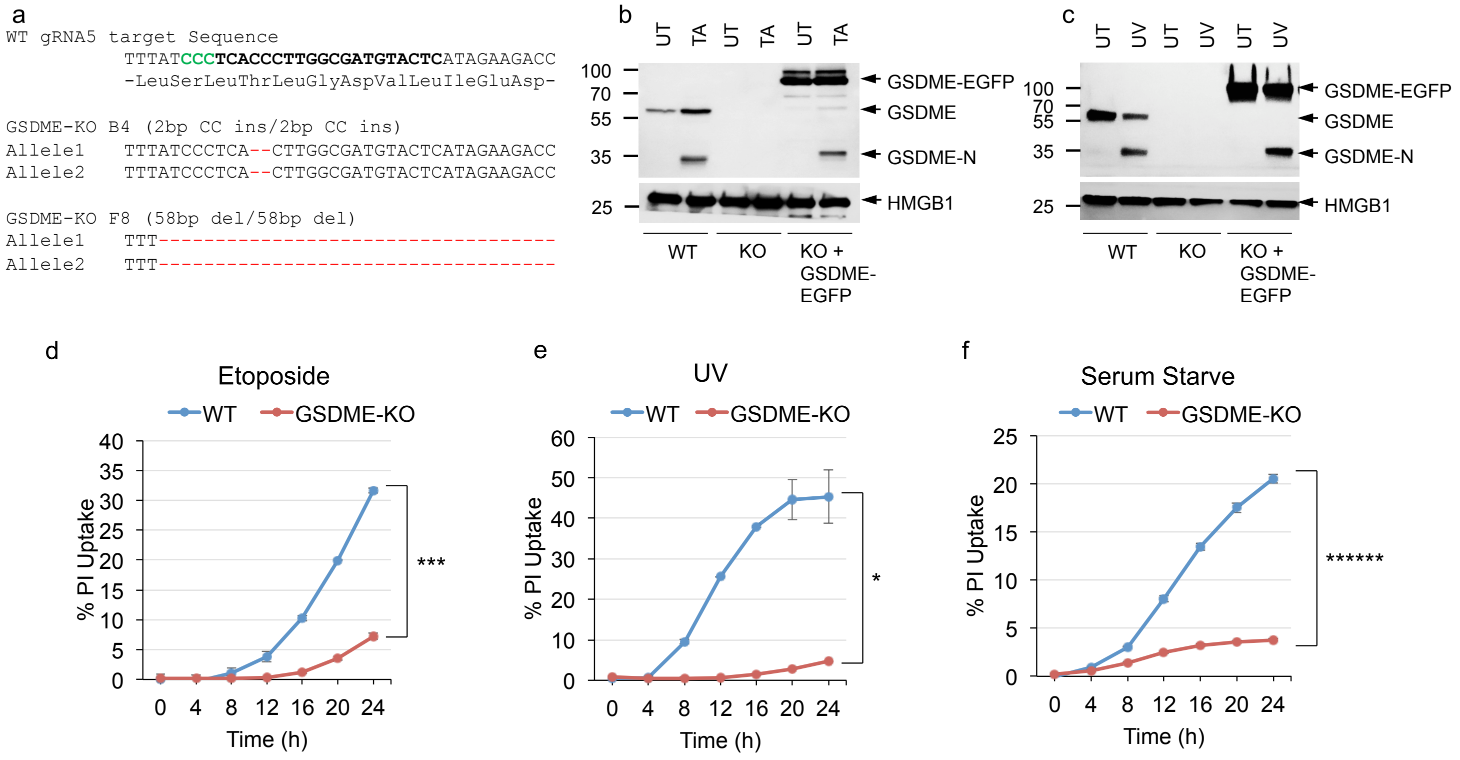

Supplementary Figure 1: Knockout of GSDME in CEM-C7 cells. (a) DNA sequence of the gRNA-targeted exon 2 in the human GSDME locus. The gRNA5-targeted sequence is highlighted in bold the PAM is highlighted in green. Deleted bases in two representative knockout clones of CEM-C7 (B4, F8) cells are indicated by red hyphens. GSDME-KO B4 clone was used in all subsequent studies. (**b**, **c**) Immunoblots of GSDME and HMGB1 in cell lysates of WT, GSDME-KO (KO), and GSDME-EGFP-reconstituted GSDME-KO (KO + GSDME-EGFP) CEM-C7 cells untreated (UT) or treated with TA (**a**) or UV (**b**) as indicated. (**d-e**) PI-uptake in WT and GSDME-KO CEM-C7 cells treated with Etoposide (**d**), UV (**e**), or serum starvation (**f**) as measured on the IncuCyte over time. Error bars represent S.D. Student's t-test, **p*<0.05, ****p*<0.0005, *******p*<0.0000005. Source data are provided as a Source Data file.

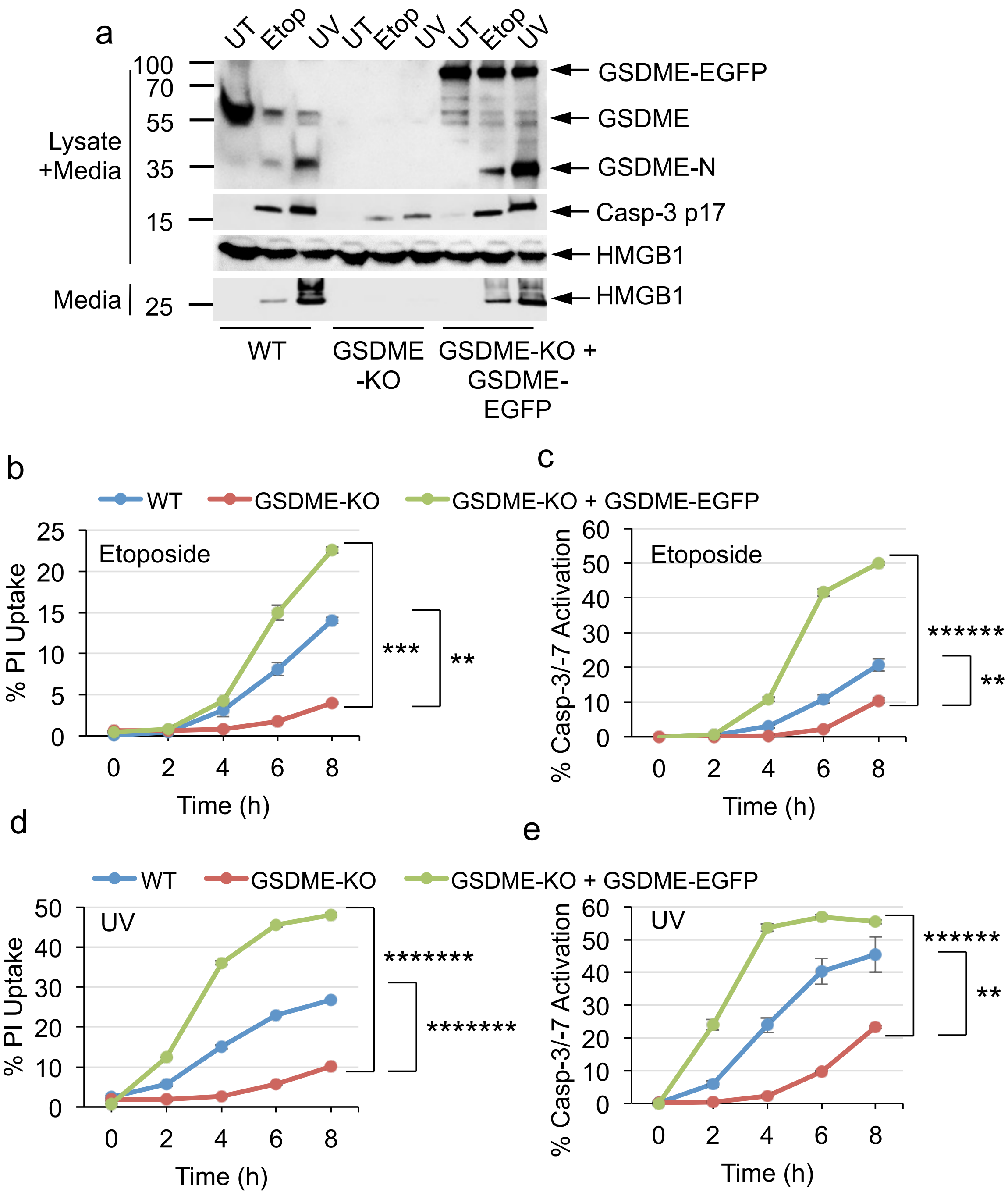

Supplementary Figure 2: Knockout of GSDME in iBMDMs compromises caspase-3 activation and pyroptosis. (a) Immunoblots of GSDME, active caspase-3 p17 and HMGB1 in combined cell lysates plus culture media of WT, GSDME-KO, and GSDME-EGFP-reconstituted GSDME-KO (GSDME-KO + GSDME-EGFP) iBMDMs untreated (UT) or treated with etoposide (Etop) or UV as indicated. (**b-e**) PI-uptake (**b**, **d**), and active caspase-3 staining (**c**, **e**) in WT, GSDME-KO, and GSDME-EGFP-reconstituted GSDME-KO (GSDME-KO + GSDME-EGFP) iBMDMs treated with etoposide (**b**, **c**) or UV (**d**, **e**) as measured on the IncuCyte over time. Error bars represent S.D. Student's t-test, ***p*<0.005, ****p*<0.0005, *******p*<0.0000005, ********p*<0.00000005. Source data are provided as a Source Data file.

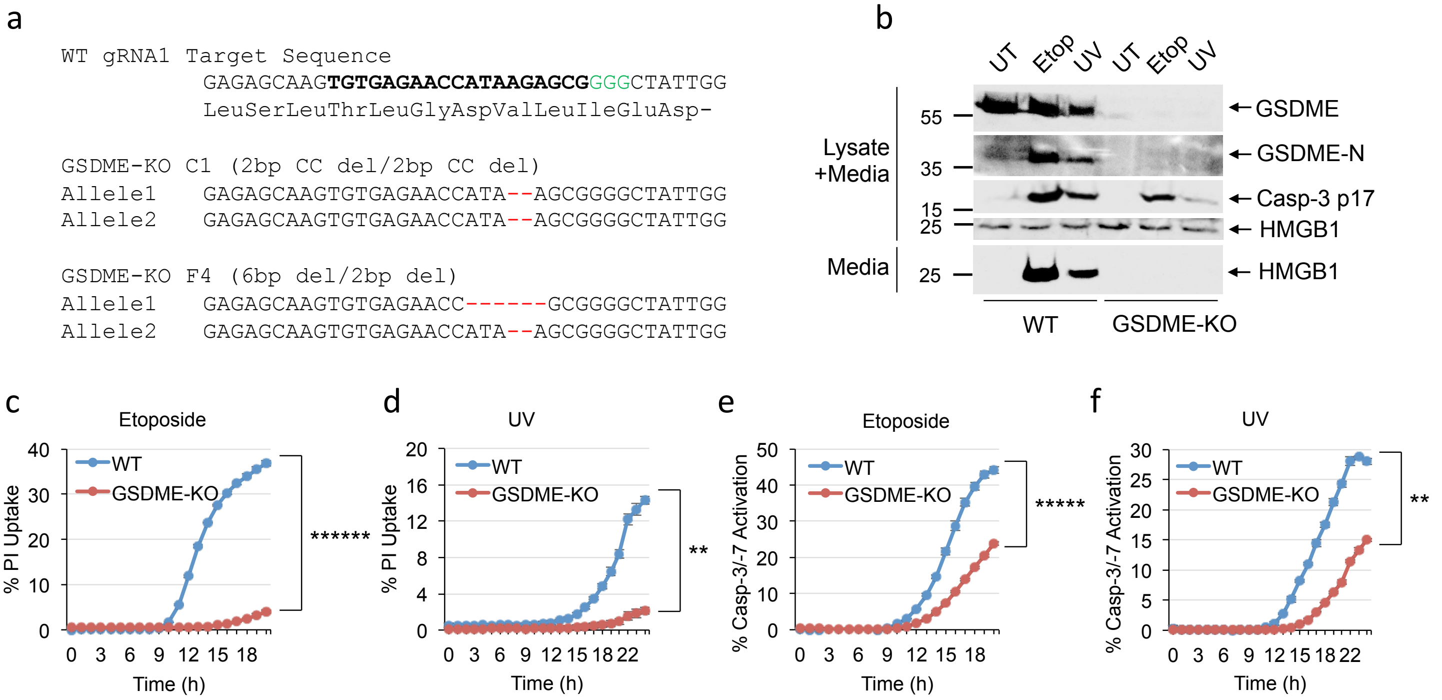

Supplementary Figure 3: Knockout of GSDME in B16-Ova cells compromises caspase-3 activation and pyroptosis. (a) DNA sequence of the gRNA-targeted exon 2 in the mouse GSDME locus. The gRNA2-targeted sequence is highlighted in bold the PAM is highlighted in green. Deleted bases in two representative knockout clones of B16-Ova (C1, F4) cells are indicated by red hyphens. GSDME-KO F4 clone was used in all studies. **(b)** Immunoblots of GSDME, active caspase-3 p17 and HMGB1 in combined cell lysates plus culture media of WT and GSDME-KO, B16-Ova cells untreated (UT) or treated with etoposide (Etop) or UV as indicated. (**c-f**) PI-uptake (**c, d**), and active caspase-3 staining (**e, f**) in WT and GSDME-KO B16-Ova cells treated with etoposide (**c**, **e**) or UV (**d**, **f**) as measured on the IncuCyte over time. Error bars represent S.D. Student's t-test, **p<0.005, *****p<0.000005, ******p<0.0000005. Source data are provided as a Source Data file.

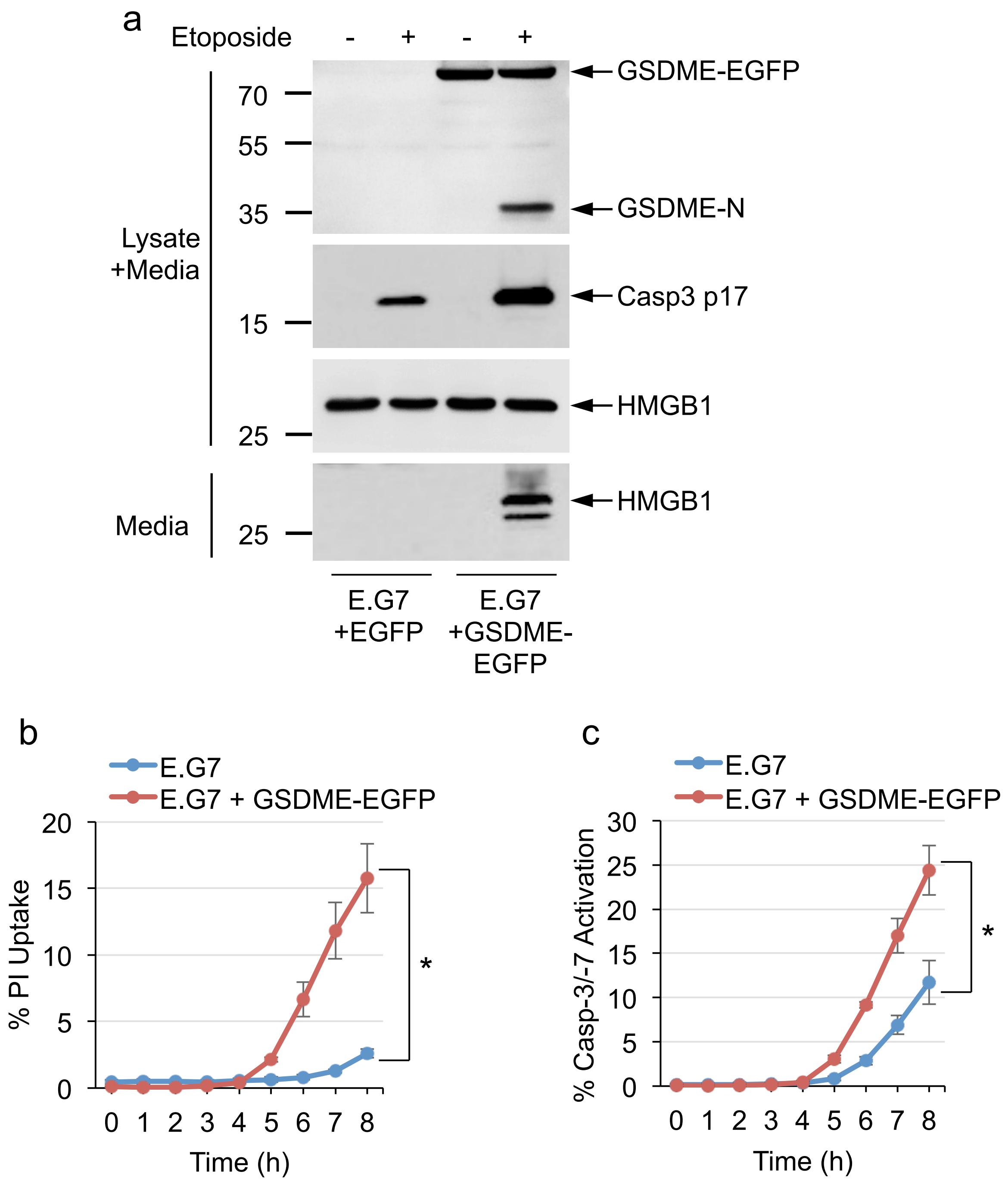

Supplementary Figure 4: Expression of GSDME in GSDME-deficient EG7-Ova promotes caspase-3 activation and pyroptosis. (a) Immunoblots of GSDME, active caspase-3 p17 and HMGB1 in combined cell lysates plus culture media of EGFP- or GSDME-EGFP-reconstituted EG7-Ova cells untreated (-) or treated with etoposide (+) as indicated. (**b**, **c**) PI-uptake (**b**), and active caspase-3 staining (**c**) in EGFP- or GSDME-EGFP-reconstituted EG7-Ova cells treated with etoposide as measured on the IncuCyte over time. Error bars represent S.D. Student's t-test, *$p<0.05$. Source data are provided as a Source Data file.

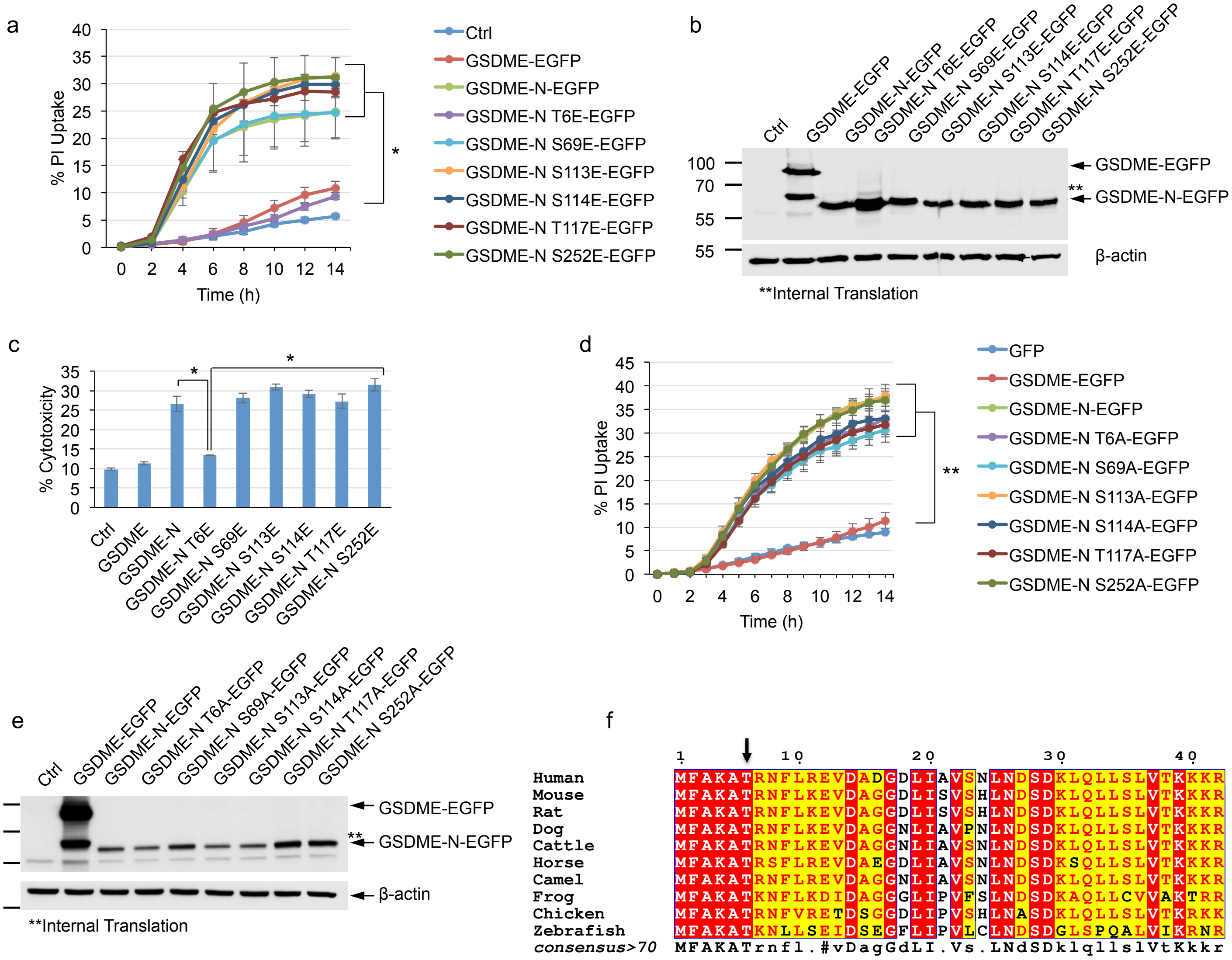

Supplementary Figure 5: Effect of phosphomimetic and alanine mutations on the pyroptotic activity of GSDME. (**a**, **c**) Pyroptosis in 293T cells transfected with WT or the indicated phosphomimetic S/T to E GSDME-N-EGFP constructs as measured by PI uptake on the IncuCyte over time (**a**) or LDH release 20 h after transfection (**c**). (**d**) Pyroptosis in 293T cells transfected with WT or the indicated S/T to A GSDME-N-EGFP constructs as measured by PI uptake on the IncuCyte over time. EGFP and full-length GSDME-EGFP were used as negative controls. (**b**, **e**) Immunoblots showing the expression of the S/T to E (**b**) or S/T to A (**e**) mutant GSDME-N-EGFP fragments analyzed in **a**, **c** and **d**. Full length GSDME-EGFP is shown as a control. (**f**) Sequence alignment of GSDME from several species. The alignment was generated using T-COFFEE algorithm (http://tcoffee.crg.cat/apps/tcoffee/index.html) and presented using ESPript 3.0 (http://espript.ibcp.fr/ESPript/cgi-bin/ESPript.cgi). Identical residues are highlighted red and conserved residues are highlighted yellow. All species contain the conserved T6 residues (indicated by arrow). Error bars represent S.D. Student's t-test, **p*<0.05, ***p*<0.005. Source data are provided as a Source Data file.

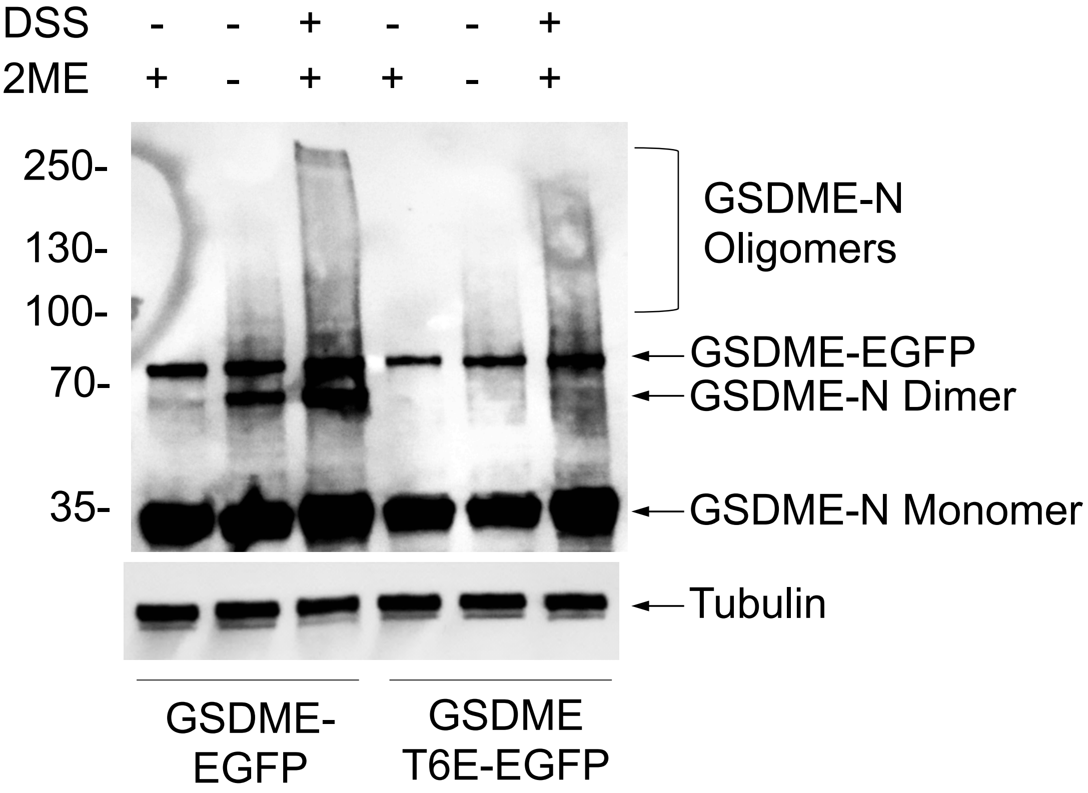

Supplementary Figure 6: T6E mutation inhibits self-oligomerization of GSDME. A higher exposure of the immunoblot in Figure 2e to highlight the high molecular weight oligomers in WT GSDME-EGFP (3rd lane). A likely explanation of why the oligomers in the WT GSDME samples are not as clearly visible as the dimers is that the probability of covalently cross-linking every molecule in the oligomer is much lower than cross-linking those molecules that are more accessible in the oligomer. Therefore, more dimers will be covalently cross-linked and survive SDS-PAGE fractionation than oligomers.

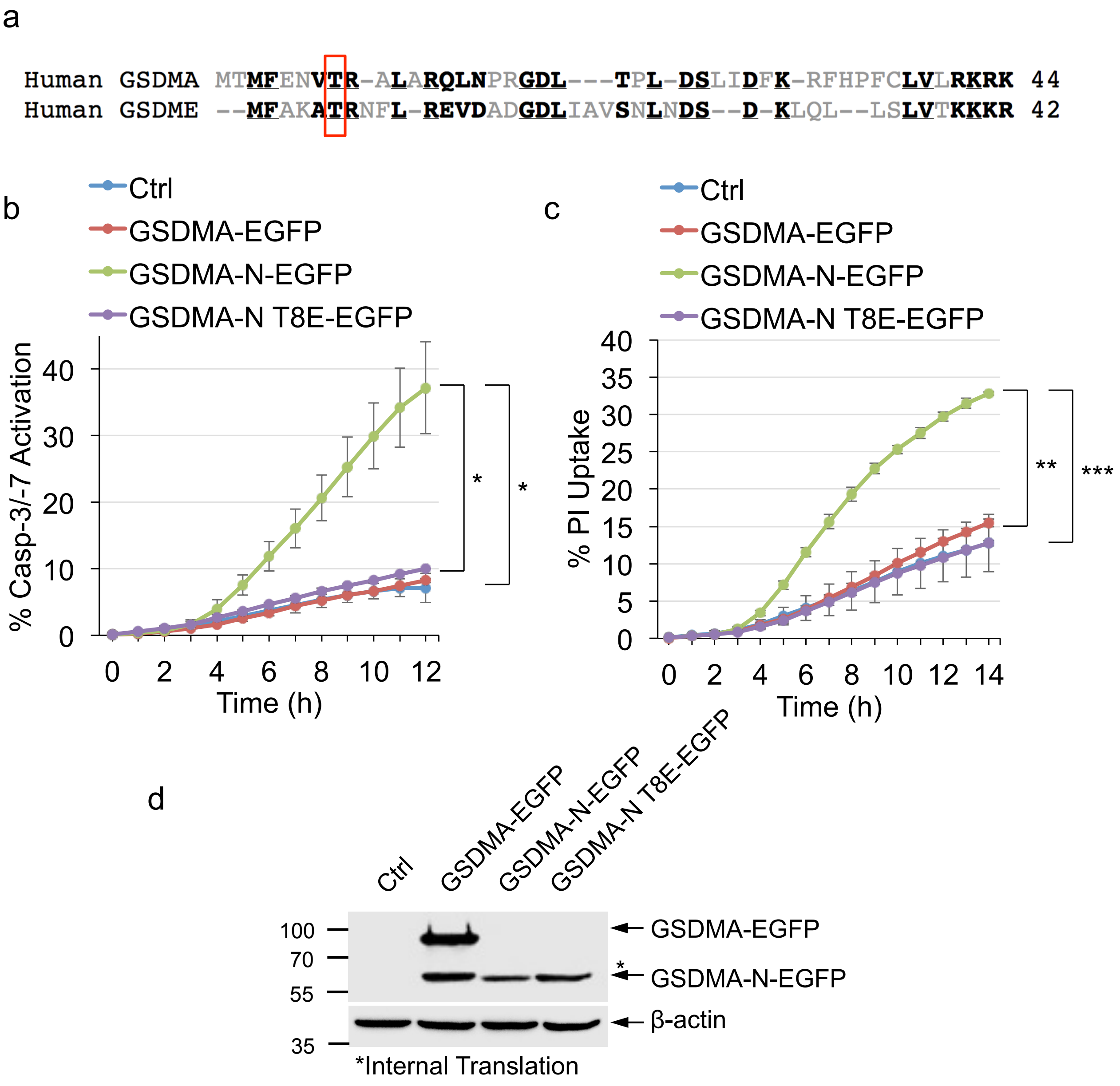

Supplementary Figure 7: Effect of phosphomimetic and alanine mutations on the pyroptotic activity of GSDMA-N. (**a**) Sequence alignment of human GSDMA and GSDME using the Sequence Manipulation Suite algorithm (http://www.bioinformatics.org/sms2/pairwise_align_protein.html). The conserved T8 of GSDMA and T6 of GSDME are indicated with a red box. (**b**, **c**) active caspase-3 staining (**b**) and PI uptake (**c**) in 293T cells transfected with full-length GSDMA-EGFP or the indicated GSDMA-N-EGFP constructs as measured on the IncuCyte over time. **(d**) Immunoblots showing the expression of the full-length GSDMA-EGFP and the WT or T8E GSDMA-N-EGFP. Error bars represent S.D. Student's t-test, *$p<0.05$, **$p<0.005$, ***$p<0.0005$. Source data are provided as a Source Data file.

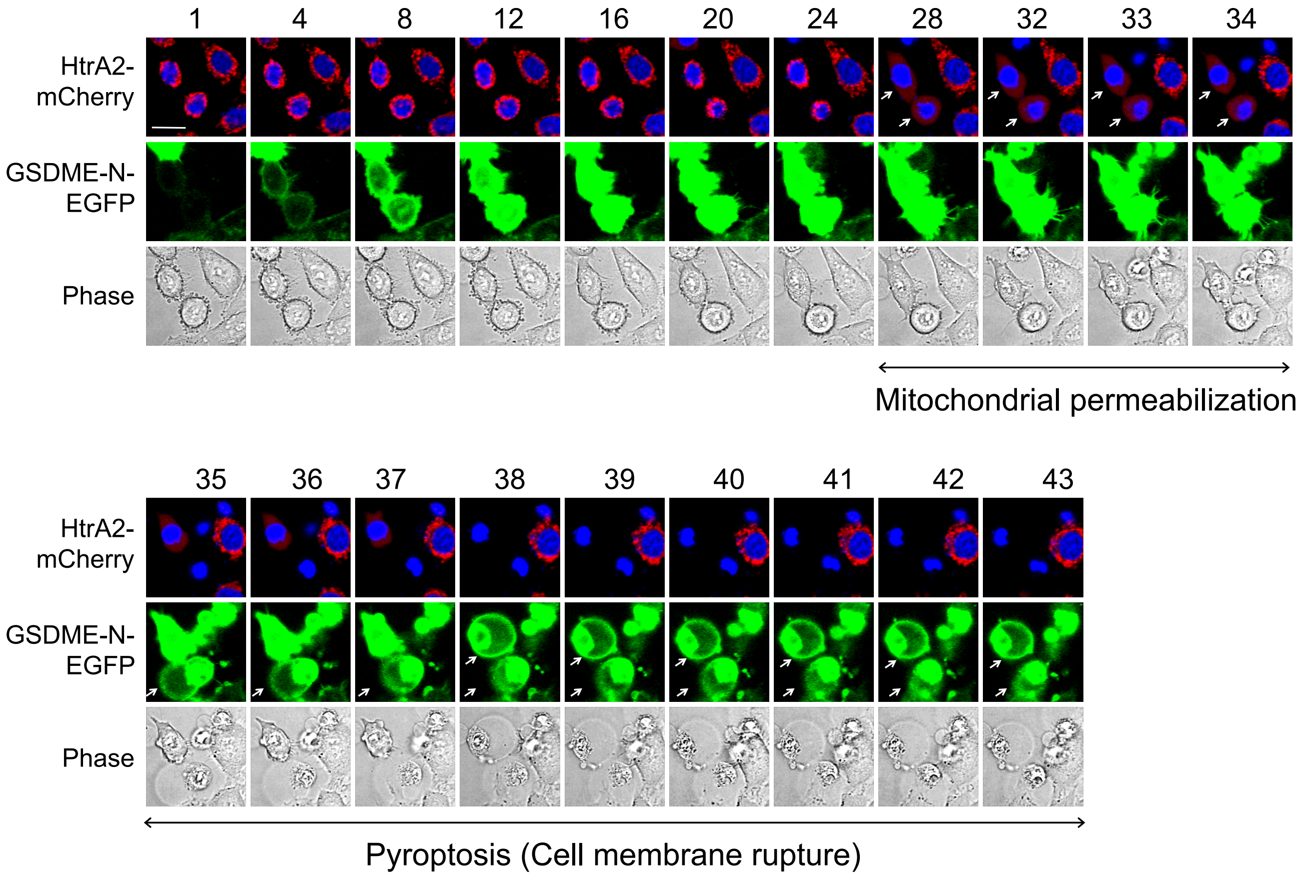

Supplementary Figure 8: GSDME-N induces mitochondrial permeabilization before rupturing the cell membrane. Time-series confocal images of GSDME-N-EGFP-transfected Hela-HtrA2-mCherry cells. Early expression of GSDME-N-EGFP can be seen in images 1-4. Images were acquired every 10 min 7 hours post-transfection. The image numbers are shown above the images. The arrows in images 28-34 point to cells undergoing mitochondrial permeabilization. The arrows in images 35-43 point to cells undergoing cell membrane rupture and pyroptosis. A time-lapse movie showing these events can be seen in the 'Supplementary Movie 1.' Scale bar, 20 μm.

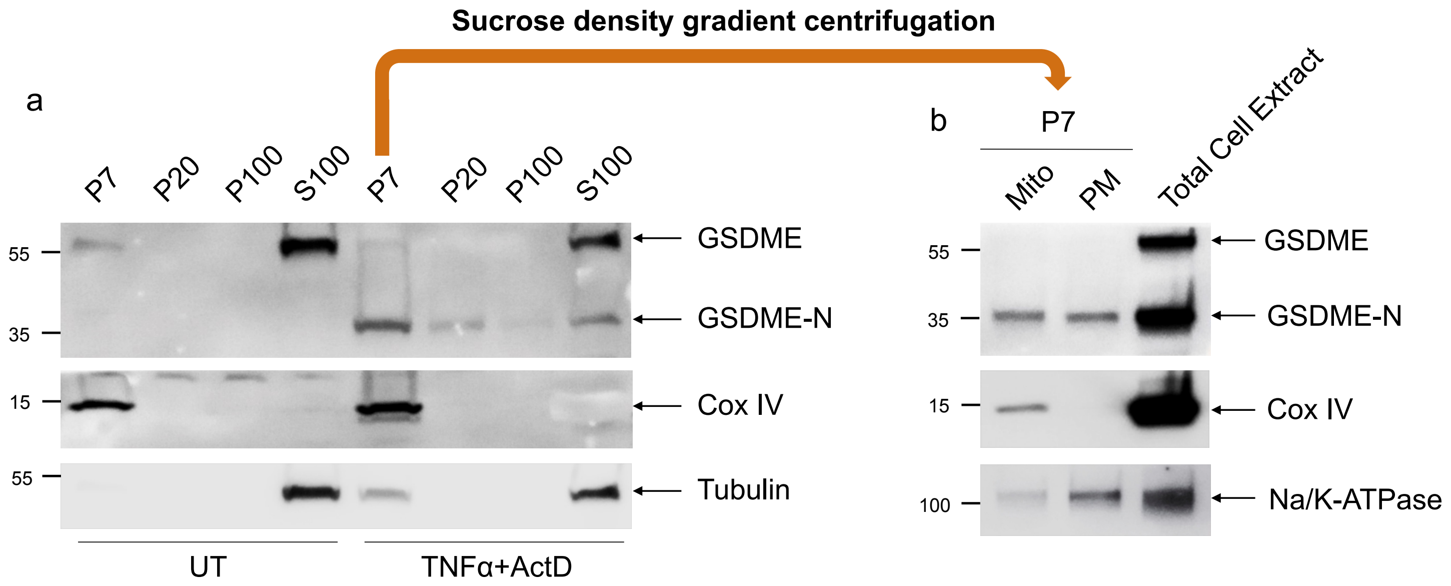

Supplementary Figure 9: GSDME-N targets the mitochondria. (a) Immunoblots of WT CEM-C7 cells untreated (UT) or treated 18 hours with TNFα+ActD and fractionated into P7 (heavy membrane), P20 (light membrane), P100 (insoluble cytosol), and S100 (soluble cytosol) and probed with anti-GSDME (upper), anti-cox IV (mitochondrial marker; middle), or anti-tubulin antibodies (cytosolic marker; lower). **(b)** Immunoblots of the P7 fraction of WT CEM-C7 cells treated for 18 hours with TNFα+ActD and further fractionated on a sucrose step density gradient to separate the plasma membrane (PM) and mitochondria (mito). The purified PM and Mito fractions were probed with anti-GSDME (upper), anti-cox IV (mitochondrial marker; middle), or Na/K-ATPase (plasma membrane marker; lower).

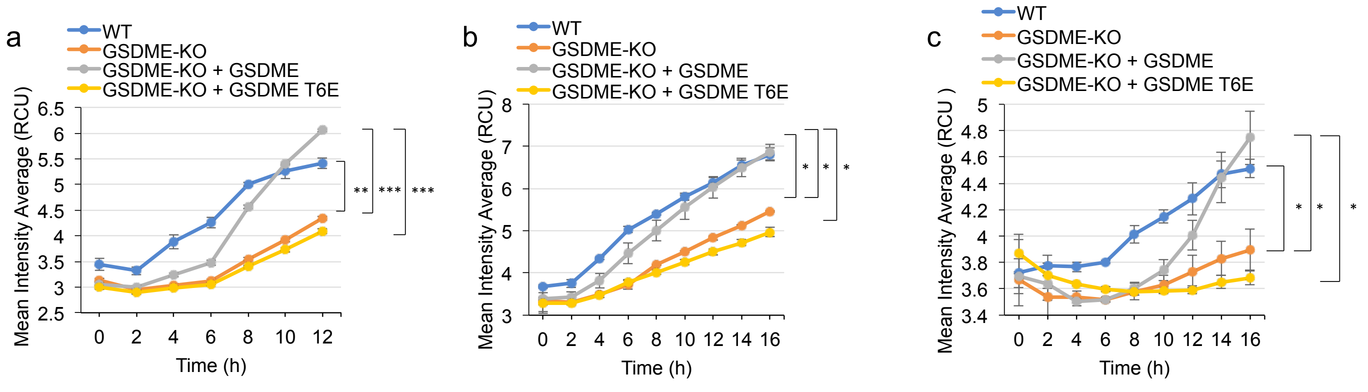

Supplementary Figure 10: GSDME-induced mitochondrial permeabilization increases ROS production in CEM-C7 cells. (**a**-**c**) Cellular ROS generation was measured in wild-type (WT), GSDME-knockout (GSDME-KO), or GSDMEKO reconstituted with WT GSDME-EGFP (GSDME-KO + GSDME) or GSDME-T6E-EGFP (GSDME-KO + GSDMET6E) CEM-C7 cells after treatment with etoposide (**a**), UV irradiation (**b**), and TNFα (**c**) as measured by an increase in the fluorescence intensity of the CellROX stain and recorded on the IncuCyte over time. Error bars represent S.D. Student's t-test, *p<0.05, **p<0.005, ***p<0.0005. Source data are provided as a Source Data file.

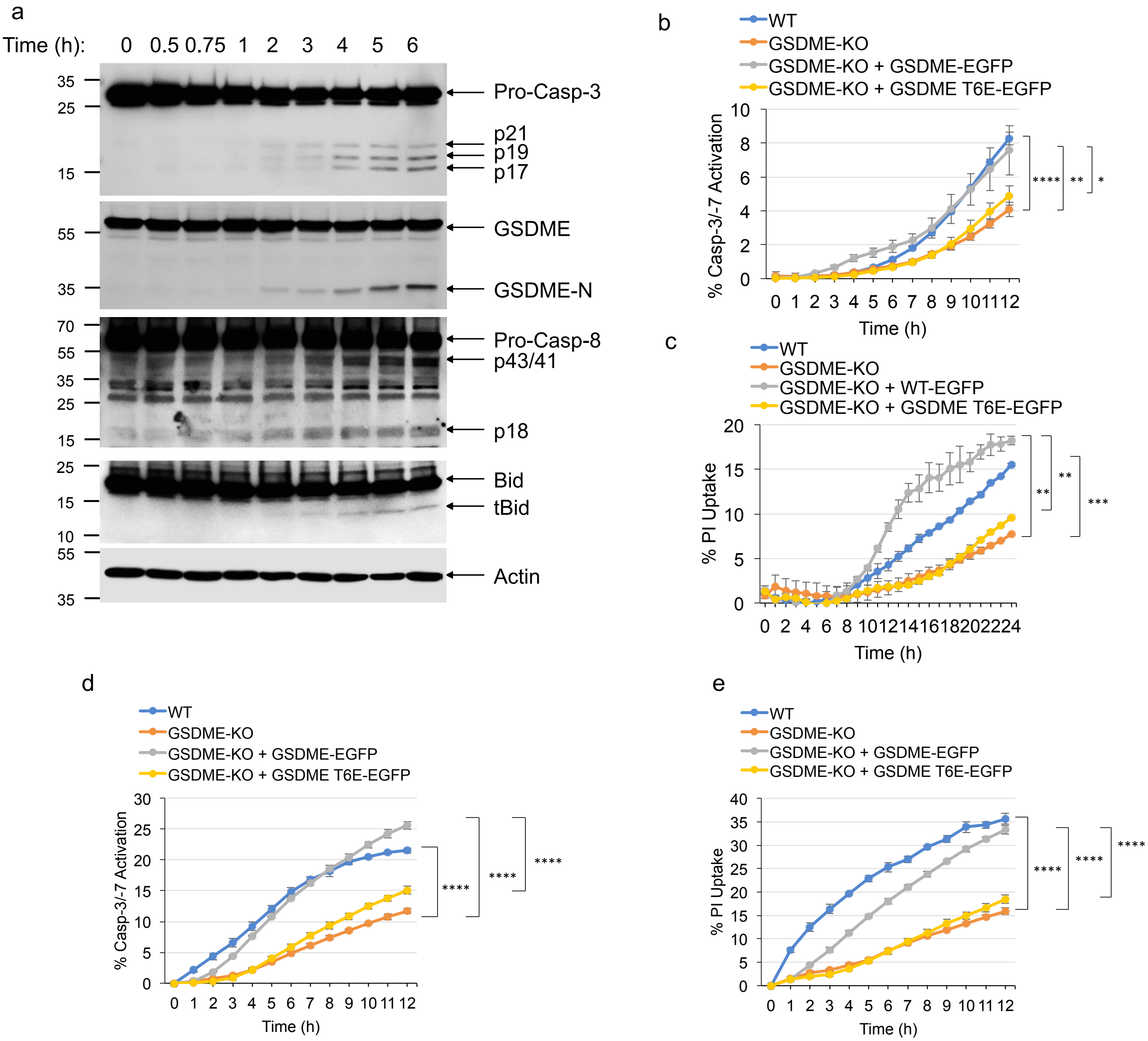

Supplemental Figure 11. GSDME augments death-receptor-induced pyroptosis and caspase-3 activation. (a) Immunoblots examining activation of pro-caspase-3 and -8, and their respective substrates, GSDME and Bid, in WT CEM-C7 cells after treatment with TNFα + ActD over the course of 6 hours. Actin serves as a loading control. **(b-e)** Caspase-3/-7 activation (**b, d**) and PI uptake (**c, e**) were monitored in CEM-C7 cells (WT, GSDME-KO, or GSDME-KO reconstituted with WT GSDME-EGFP or GSDME-T6E-EGFP) treated with (5Z)-7-Oxozeaenol (125 nM) + TNFα (**b, c**), or Takinib (1 µM) + TNFα (**d, e**) as measured on the IncuCyte over time. Error bars represent S.D. Student's t-test, **p*<0.05, ***p*<0.005, ****p*<0.0005, *****p*<0.00005. Source data are provided as a Source Data file.

a

```
WT gRNA target Sequence

     CCTCAGGTCAACAACGGTTCCAGCCTCAGGGATGAGTGC
     ------ValAsnAsnGlySerSerLeuArgAspGluCys

Bid-KO B11 (22bp del)
     CCTCAGGTCAACAACGGTTC-------------------

Bid-KO C4 (13bp del)
     CCTCAGGTCAACAACGGTTC-------------GAGTGC

Bid-KO E8 (11bp del)
     CCTCAGGTCAACAACGGTT-----------GATGAGTGC
```

```
WT gRNA target Sequence

     CCTCAGGTCAACAACGGTTCCAGCCTCAGGGATGAGTGC
     ------ValAsnAsnGlySerSerLeuArgAspGluCys

DKO C2 (1bp ins)
     CCTCAGGTCAACAACGGTTCCAGCCCTCAGGGATGAGTGC

DKO F7 (28bp del)
     CC----------------------------GATGAGTGC

DKO E1 (22bp del)
     CCTCAGGTCAACAACGGTTC-------------------
```

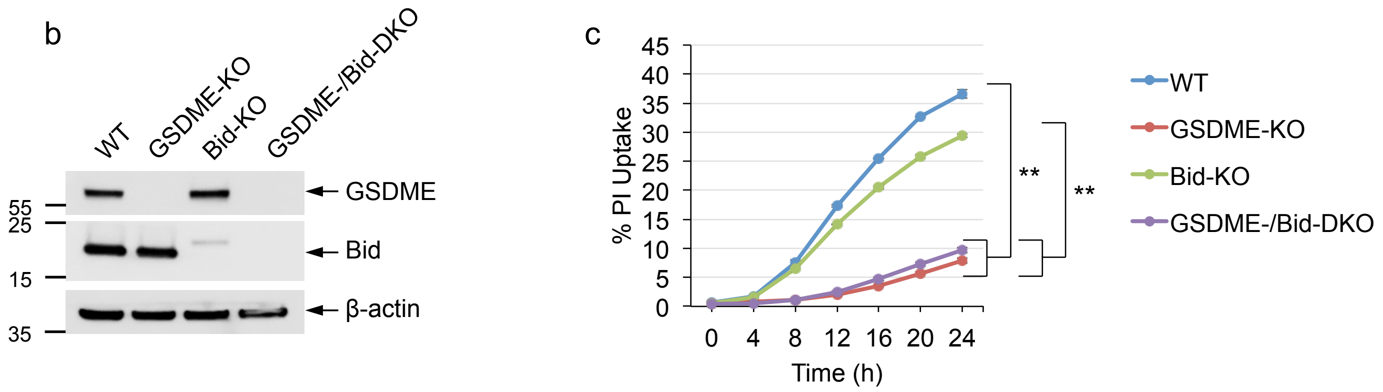

Supplementary Figure 12: Effect of Bid and GSDME deletion on TNFα-induced pyroptosis in CEM-C7 cells. (a) DNA sequences of the gRNA-targeted exon 2 in the human Bid locus. The gRNA2-targeted sequence is highlighted in bold the PAM is highlighted in green. Deleted bases in three representative Bid-KO clones (B11, C4, E8) or GSDME/Bid-dKO (C2, F7, E1) CEM-C7 cells are indicated by red hyphens. Bid-KO clone E8 and GSDME/Bid-dKO clone F7 were used in all studies. **(b)** Immunoblots of GSDME, Bid and β-actin in cell lysates of WT, GSDME-KO, Bid-KO and GSDME/Bid-dKO cells. (**c**) PI-uptake in WT, GSDME-KO, Bid-KO, and GSDME/Bid-dKO CEM-C7 cells treated with TNFα/ActD as measured on the IncuCyte over time. Error bars represent S.D. Student's t-test, ***p*<0.005. Source data are provided as a Source Data file.

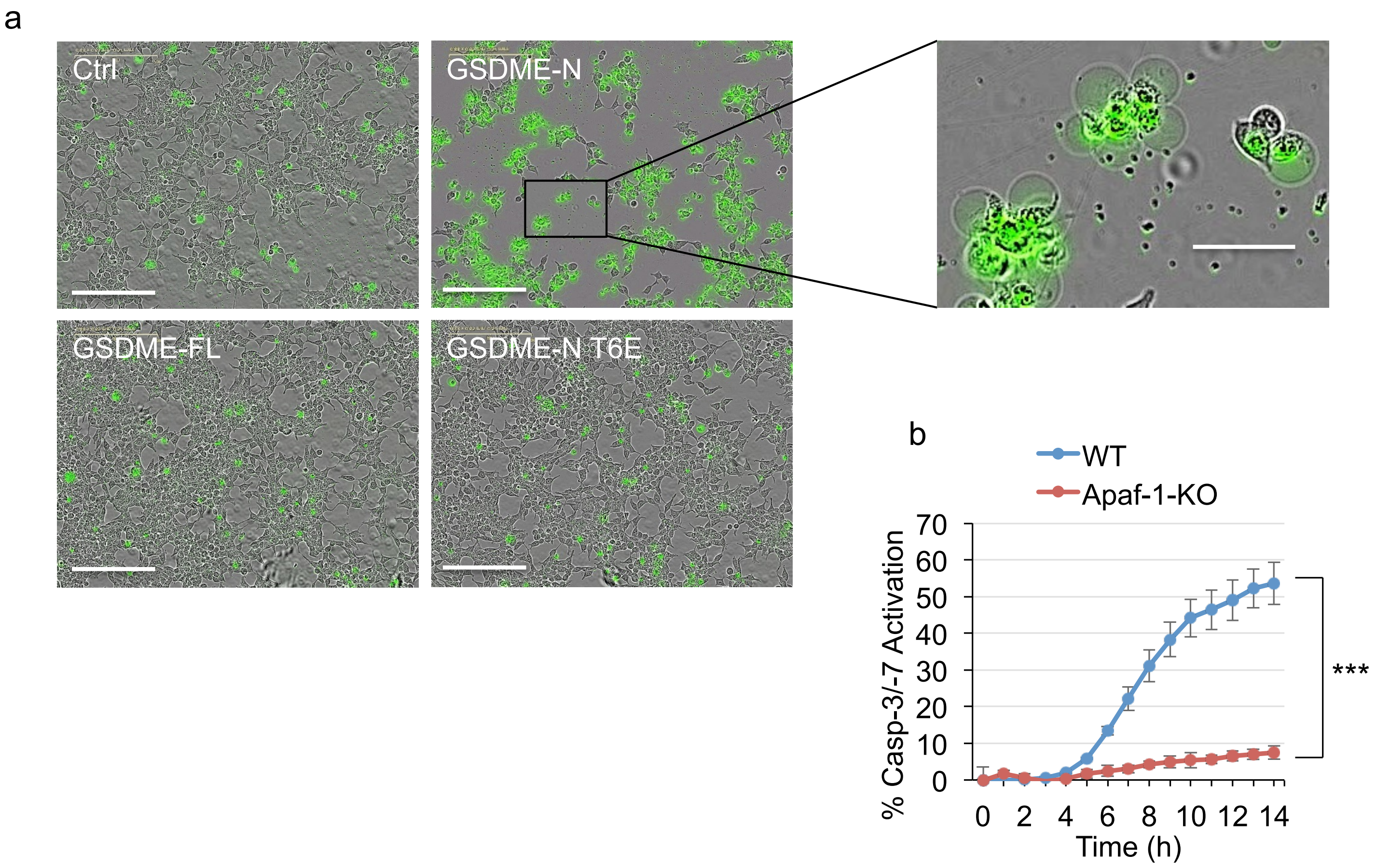

Supplementary Figure 13: GSDME-induced caspase-3 activation requires Apaf-1. **(a)** Incucyte generated images of 293T cells transfected with empty vector control (Ctrl) or the indicated GSDME constructs in the presence of the fluorescent caspase-3/-7-specific substrate (green). The inset on the right is a magnification of cells transfected with GSDME-N showing both pyroptosis and active caspase-3/-7 fluorescence. (**b**) Active caspase-3 staining in WT and Apaf-1-KO MEFs transfected with GSDME-N-EGFP construct as measured on the IncuCyte over time. Error bars represent S.D. Student's t-test, ***$p<0.0005$. Scale bar full image, 200 µm. Scale bar inset, 50 µm. Source data are provided as a Source Data file.

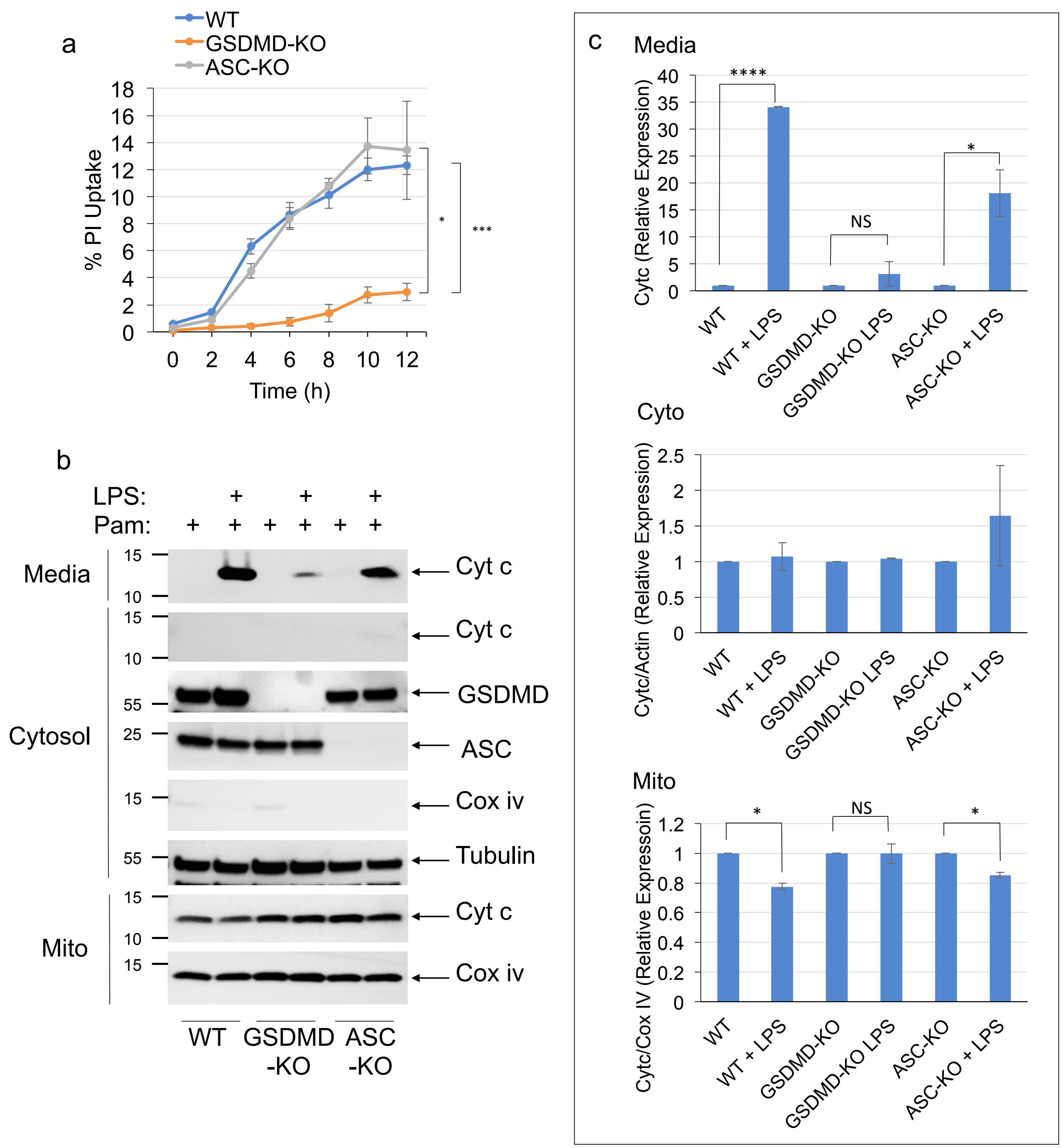

Supplementary Figure 14: GSDMD functions downstream of the inflammasome to activate caspase-3. (a) PI staining in PamCSK4-primed WT, GSDMD-KO, and ASC-KO iBMDMs transfected with LPS as measured on the IncuCyte over time. **(b)** Immunoblots of Cyt c released (1st and 2nd panels) released into culture media (media) or cytosol from WT, GSDMD-KO, or ASC-KO iBMDMs after Pam-CSK4 priming for 5 hours and LPS transfection for 12 hours. Tubulin is used as a loading control for cytosolic fraction and Cox iv is used as a loading control for mitochondrial fraction. Western blots in **b** were quantified using Image J. Values represent the mean of two independent experiments and error bars represent S.D. Student's t-test, **p*<0.05, ****p*<0.0005, *****p*<0.00005, NS, No Significance. Source data are provided as a Source Data file.

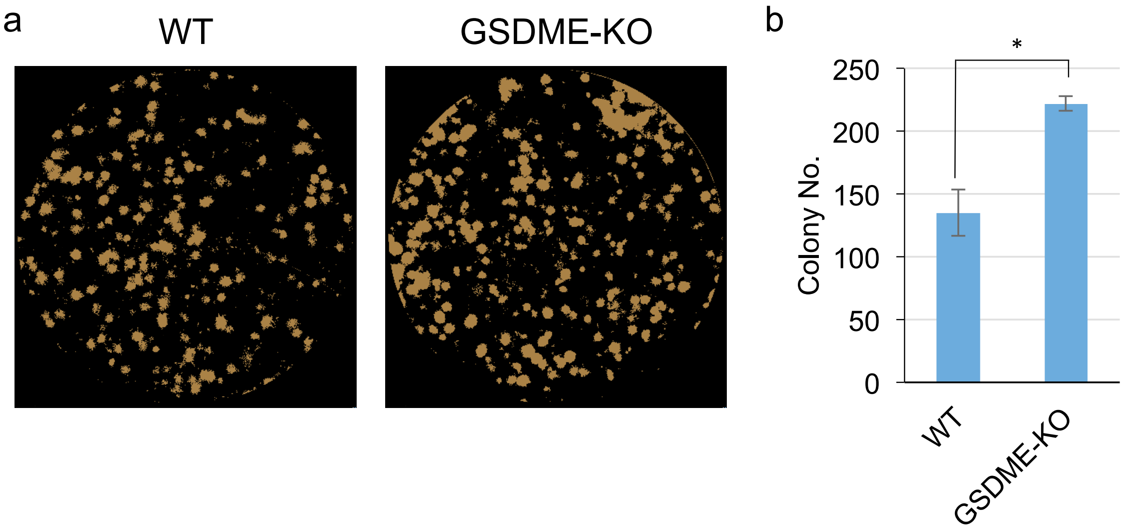

Figure 15. GSDME inhibits colony formation. WT and GSDME-KO B16-Ova cells were treated with etoposide and allowed to grow for one week. Plates were imaged on the IncuCyte and colonies were analyzed using the IncuCyte software (a) and counted (b). Values represent the mean of duplicate measurements and error bars represent S.D. Student's t-test, *,p<0.05. Source data are provided as a Source Data file.

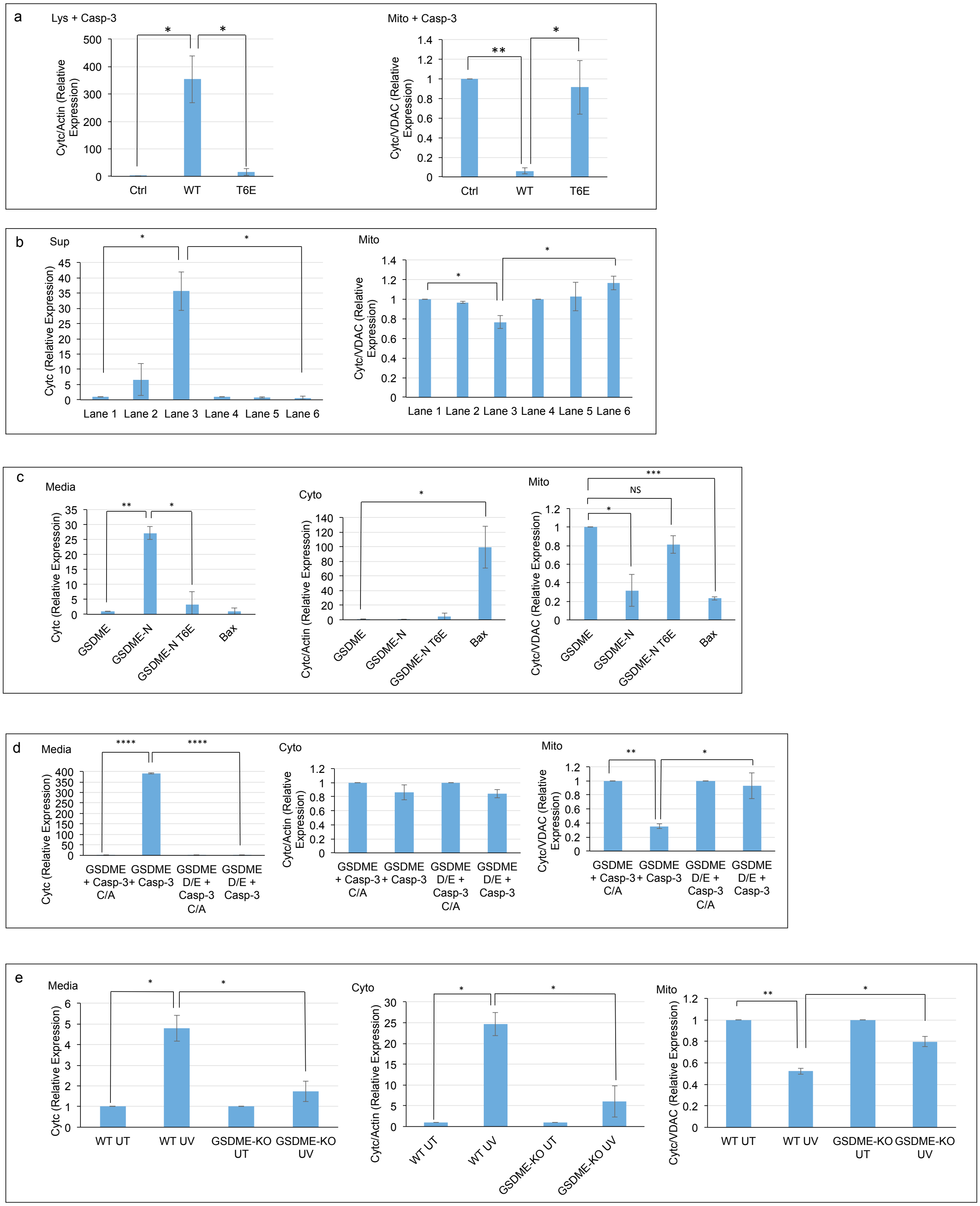

Supplementary Figure 16: Quantification of western blots in figure 5. Western blots in Figure 5a **(a)**, 5b **(b)**, 5c **(c)**, 5d **(d)**, and 5e **(e)** were quantified using Image J. Values represent the mean of two independent experiments and error bars represent S.D. Student's t-test, **p*<0.05, ***p*<0.005 ****p*<0.0005, *****p*<0.00005, NS, No Significance. Source data are provided as a Source Data file.

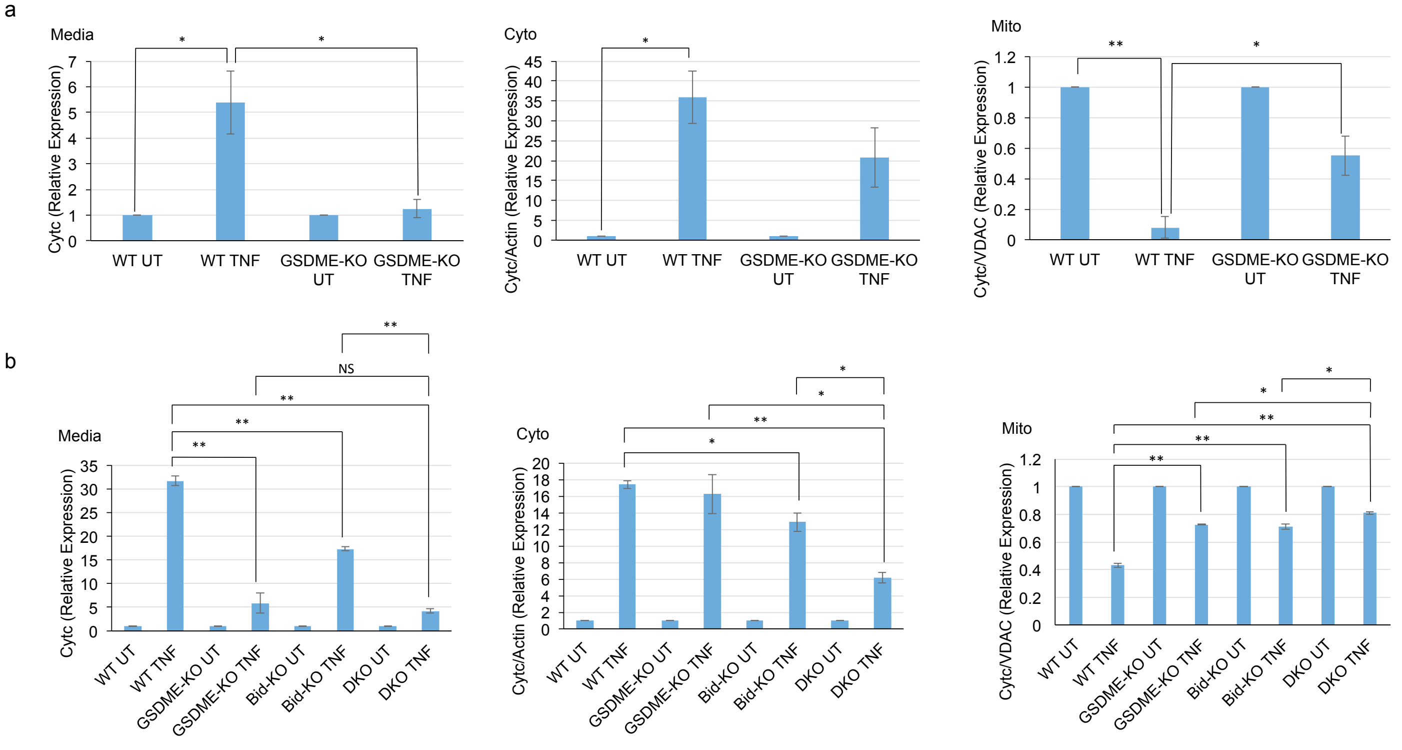

Supplementary Figure 17: Quantification of western blots in figure 6. Western blots in Figure 6d **(a)** and 6f **(b)** were quantified using Image J. Values represent the mean of two independent experiments and error bars represent S.D. Student's t-test, *p<0.05, **p<0.005**,** NS, No Significance. Source data are provided as a Source Data file.

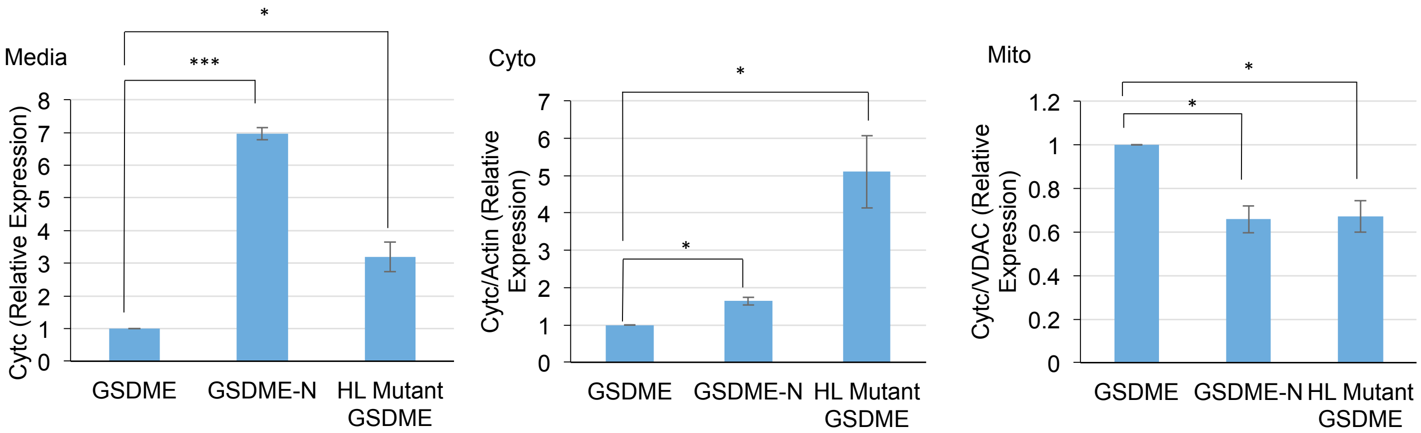

Supplementary Figure 18: Quantification of western blots in figure 7. Western blots in Figure 7f were quantified using Image J. Values represent the mean of two independent experiments and error bars represent S.D. Student's t-test, *p<0.05, ***,p<0.0005. Source data are provided as a Source Data file.

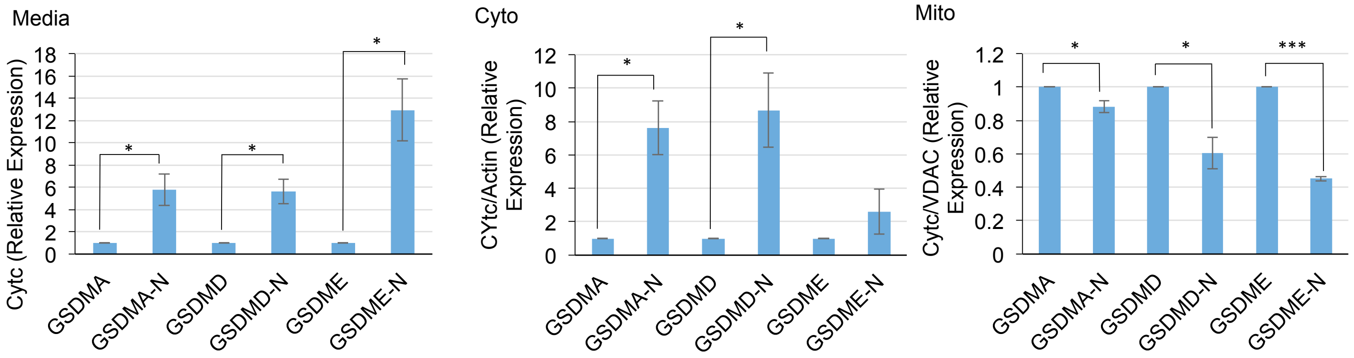

Supplementary Figure 19: Quantification of western blots in figure 8. Western blots in Figure 8d were quantified using Image J. Values represent the mean of two independent experiments and error bars represent S.D. Student's t-test, *p<0.05, ***p<0.0005. Source data are provided as a Source Data file.

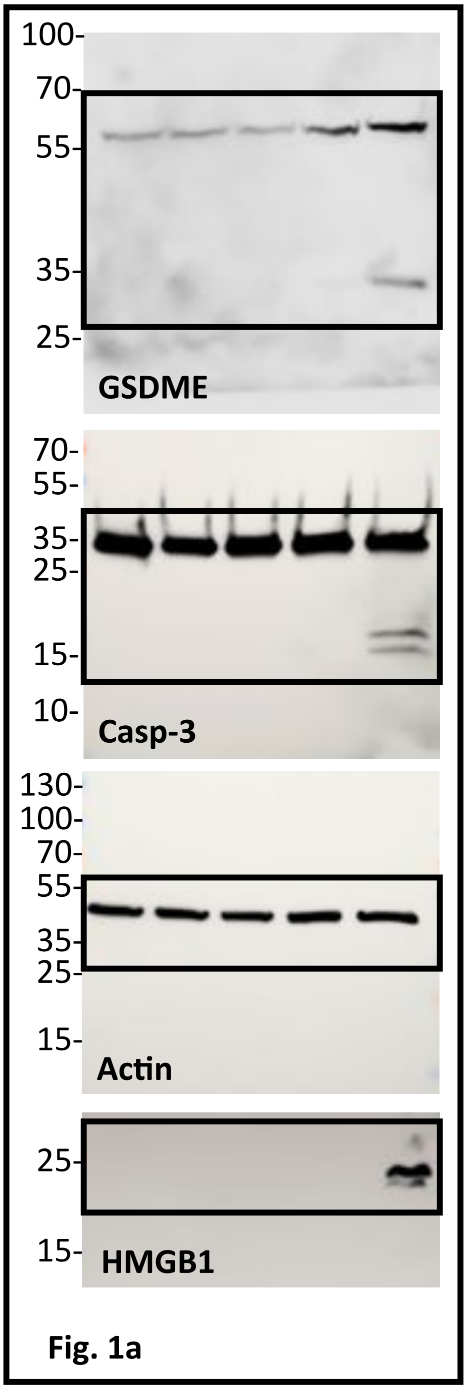

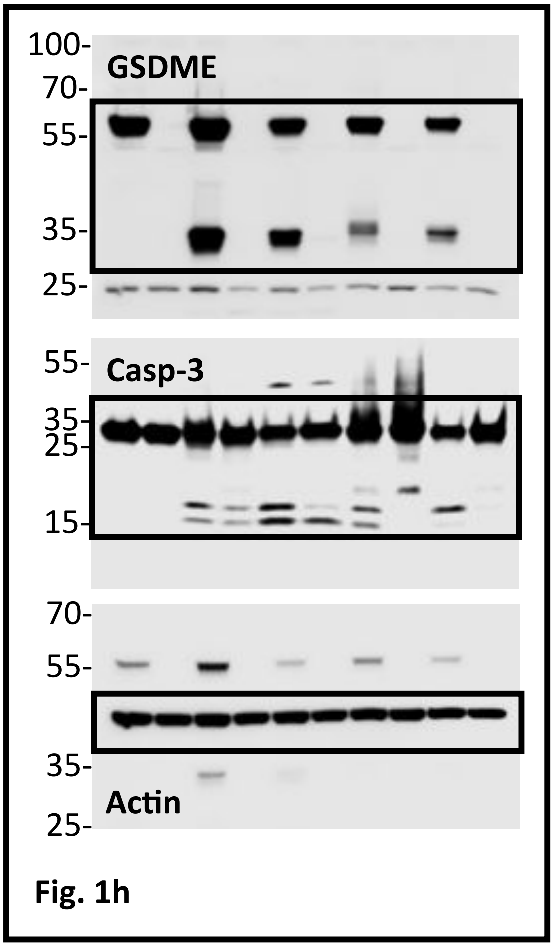

Supplementary Figure 20. Uncropped scans of western blots shown in Figure 1.

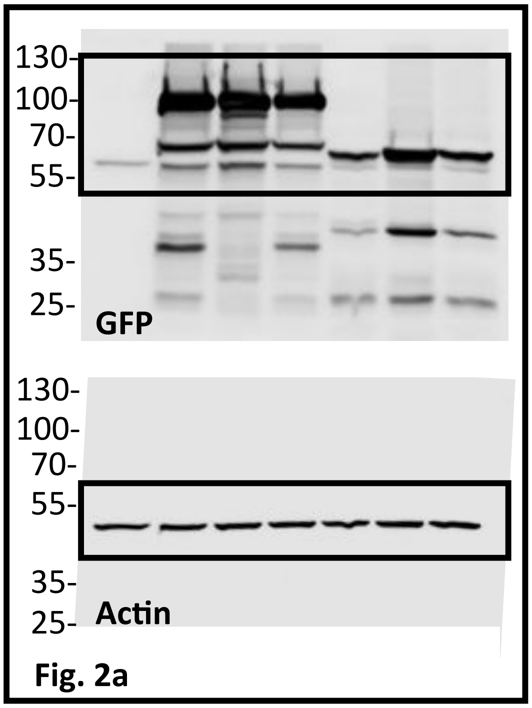

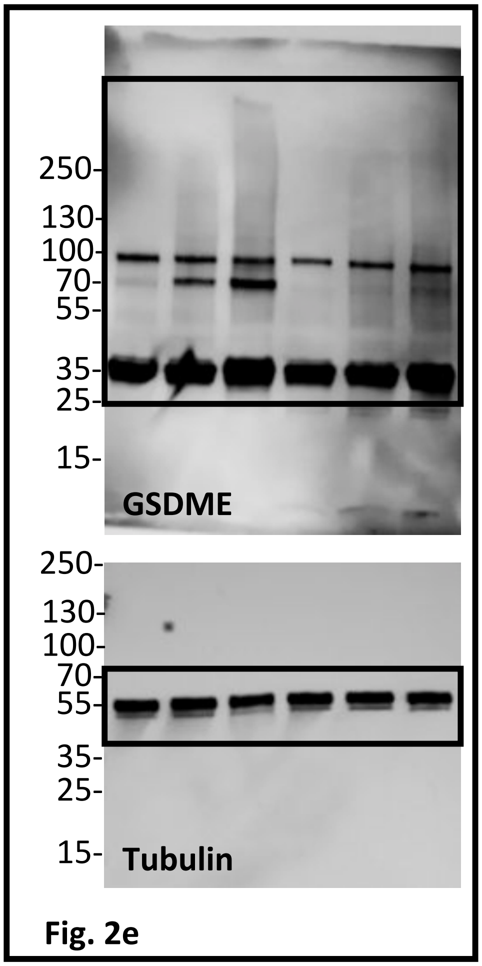

Supplementary Figure 21. Uncropped scans of western blots shown in Figure 2.

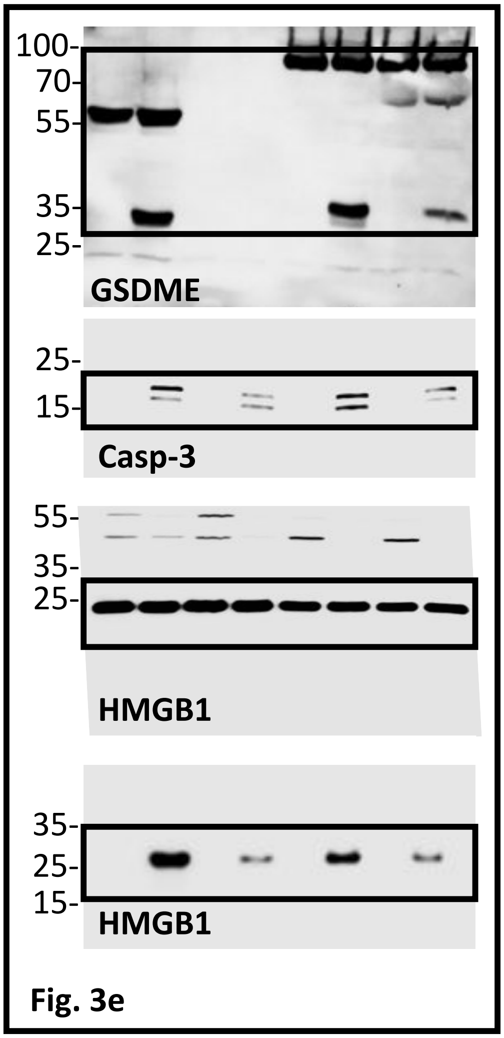

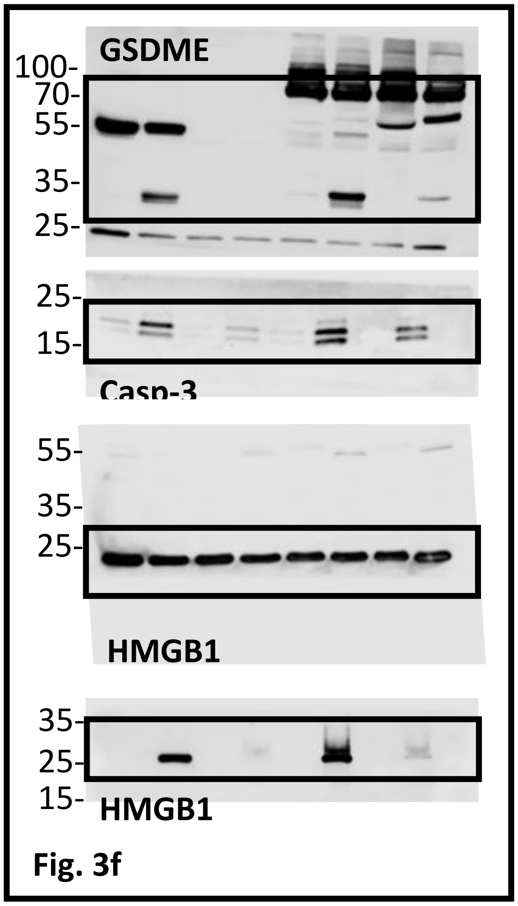

Supplementary Figure 22. Uncropped scans of western blots shown in Figure 3.

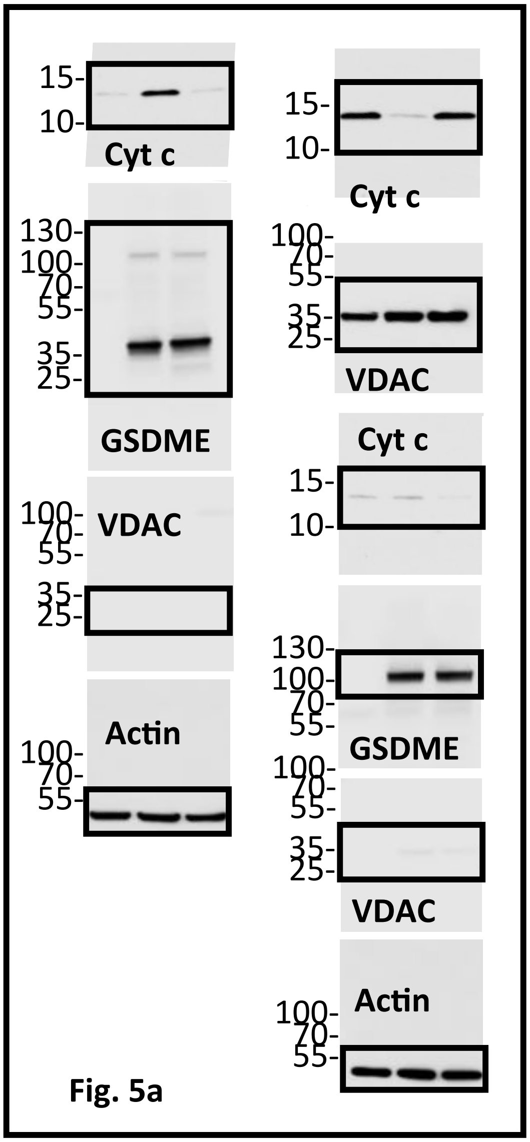

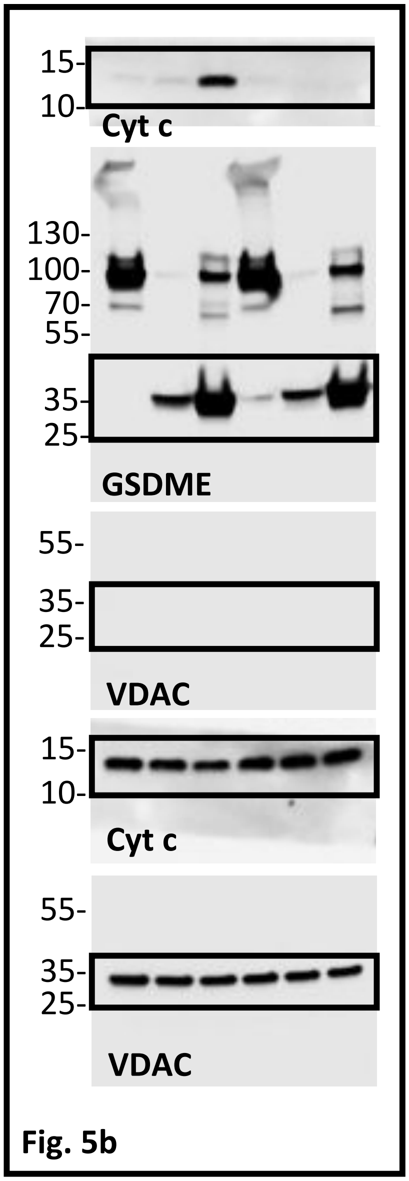

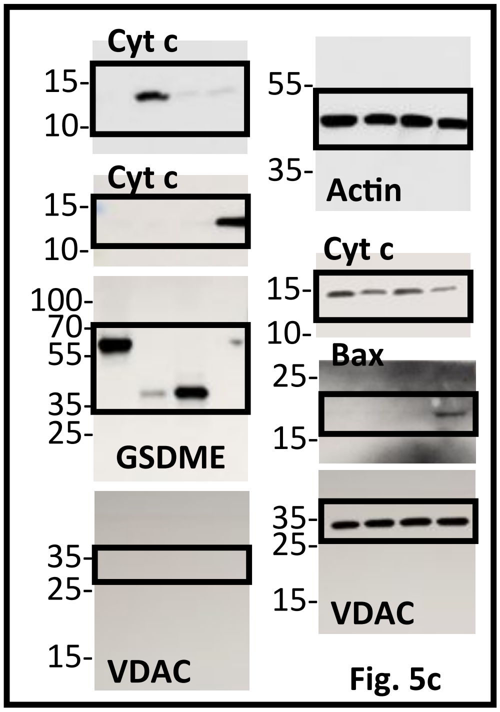

Supplementary Figure 23. Uncropped scans of western blots shown in Figure 5a-c.

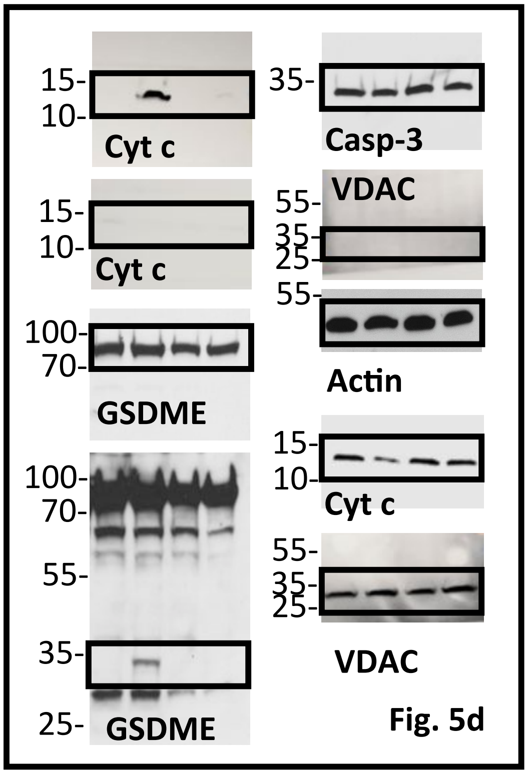

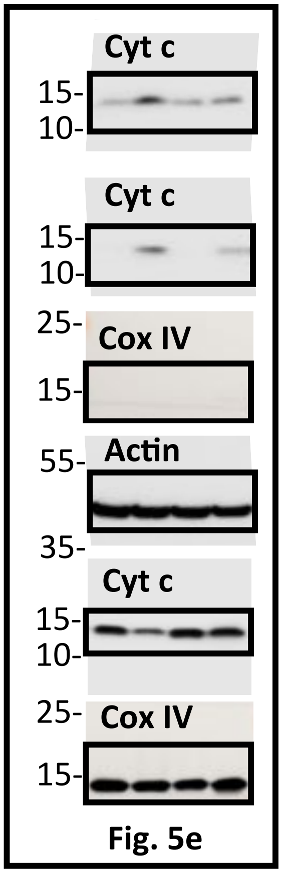

Supplementary Figure 24. Uncropped scans of western blots shown in Figure 5d-e.

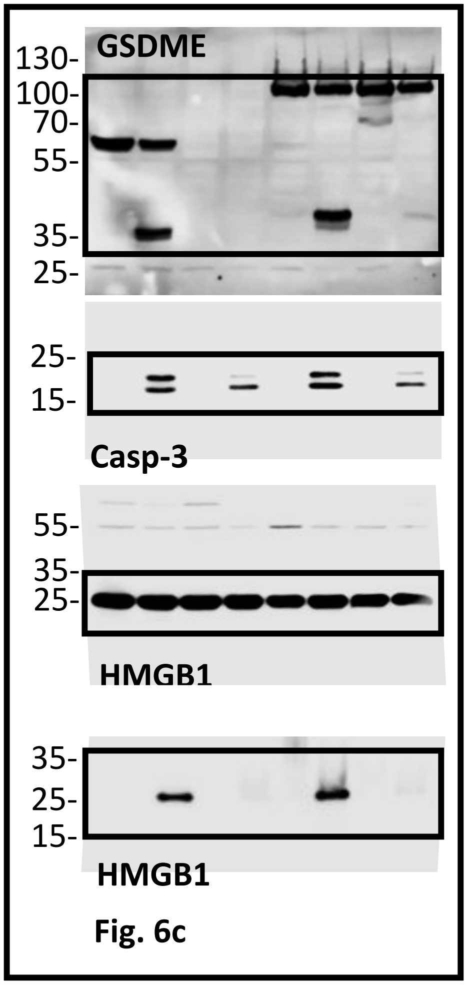

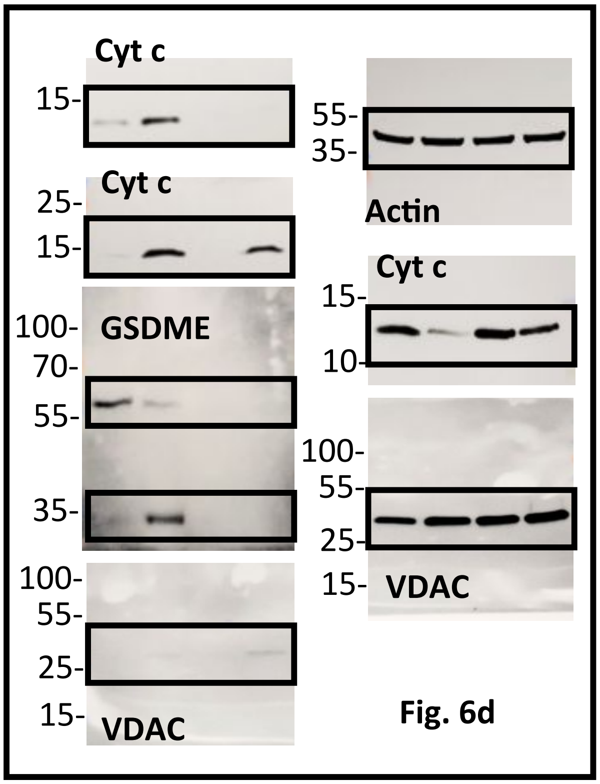

Supplementary Figure 25. Uncropped scans of western blots shown in Figure 6.

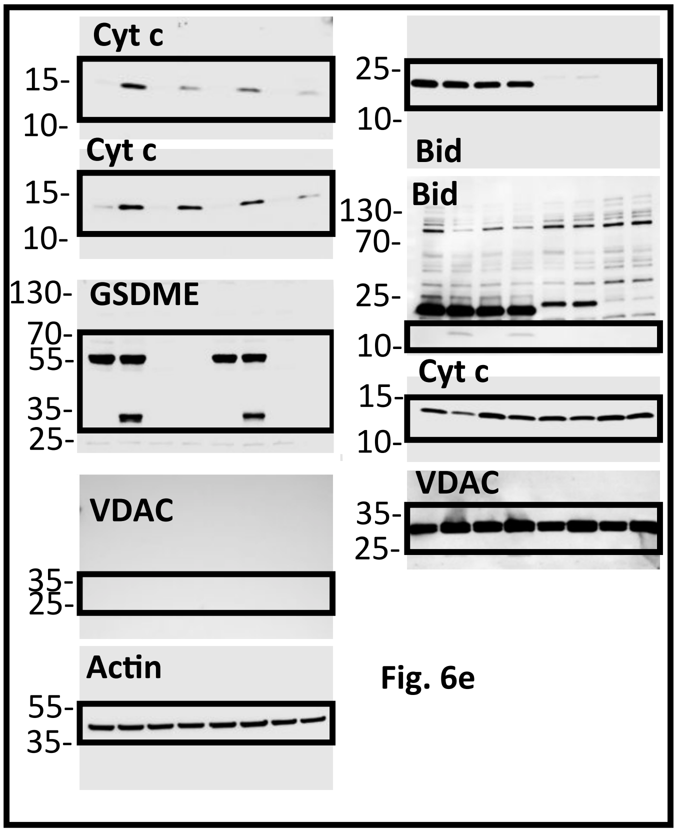

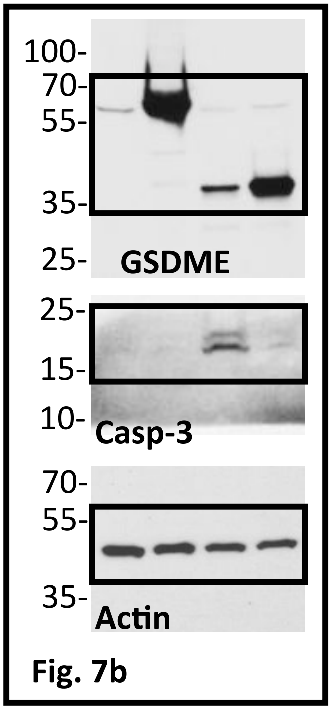

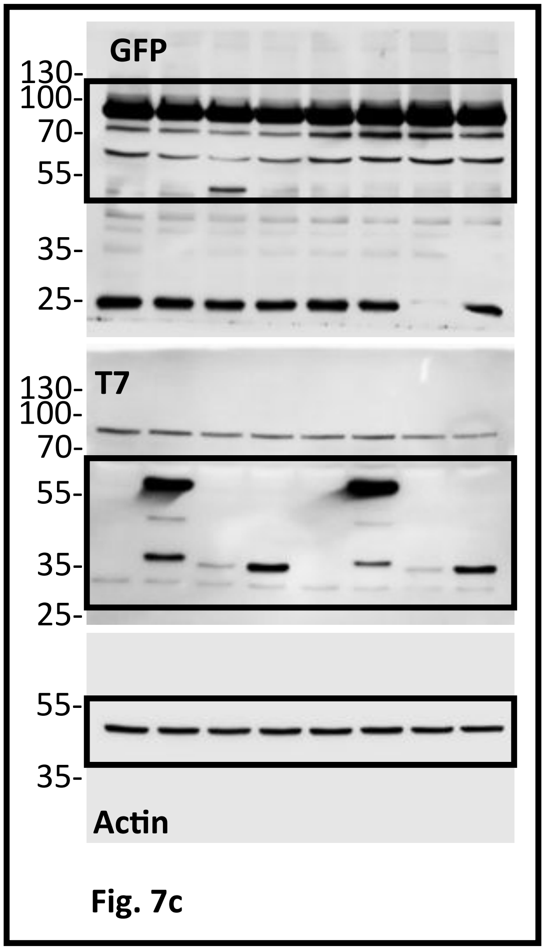

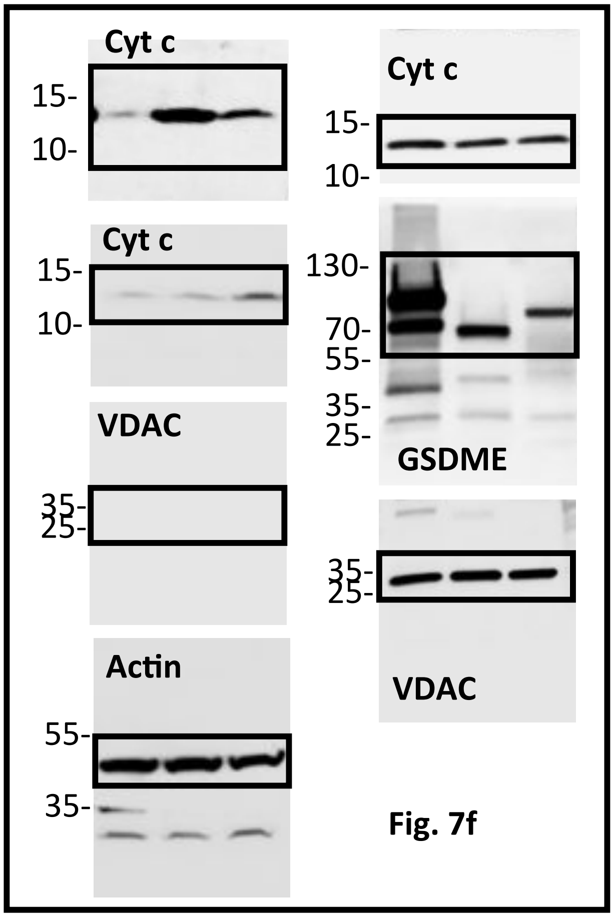

Supplementary Figure 26. Uncropped scans of western blots shown in Figure 7.

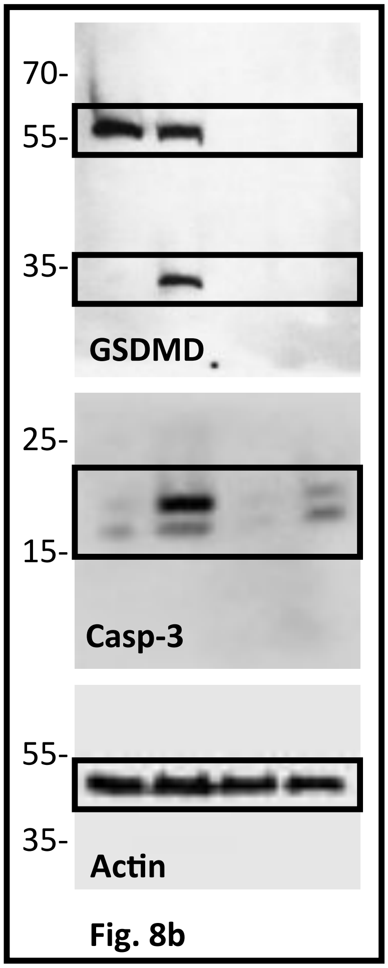

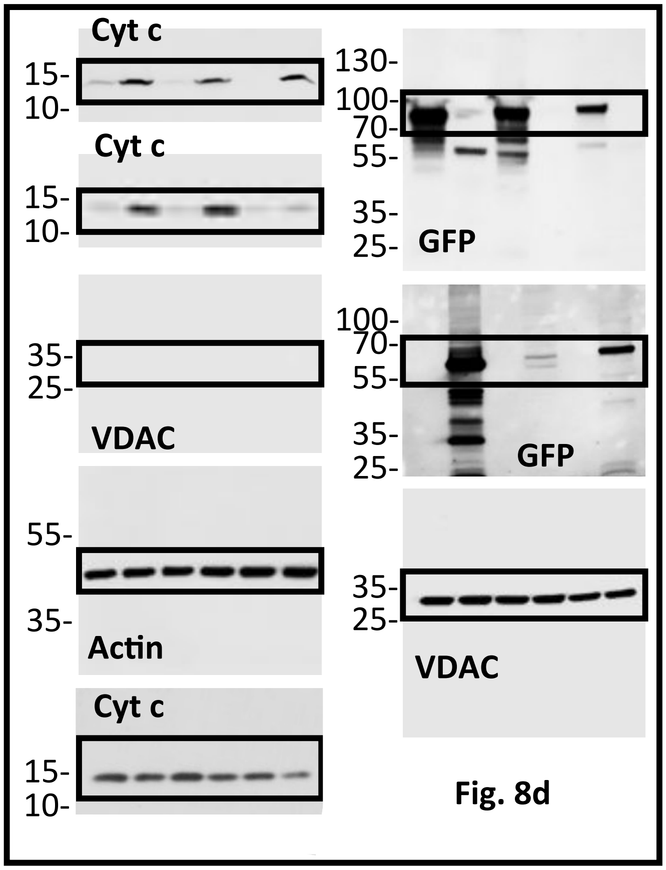

Supplementary Figure 27. Uncropped scans of western blots shown in Figure 8.

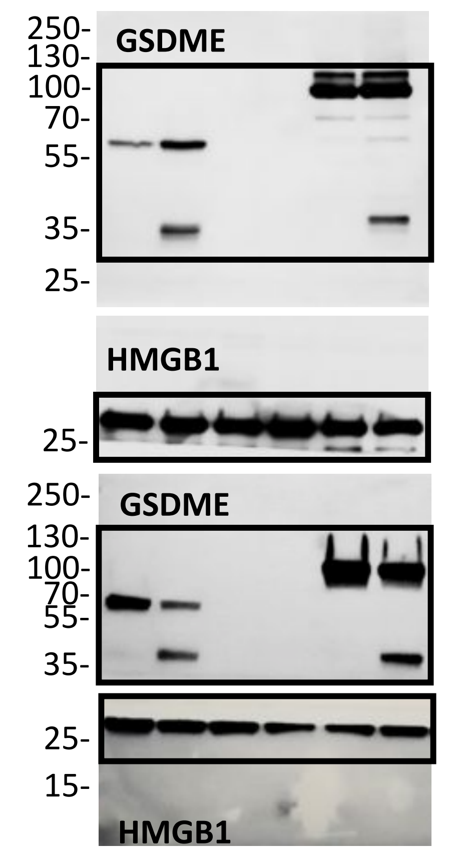

Sup. Fig. 1

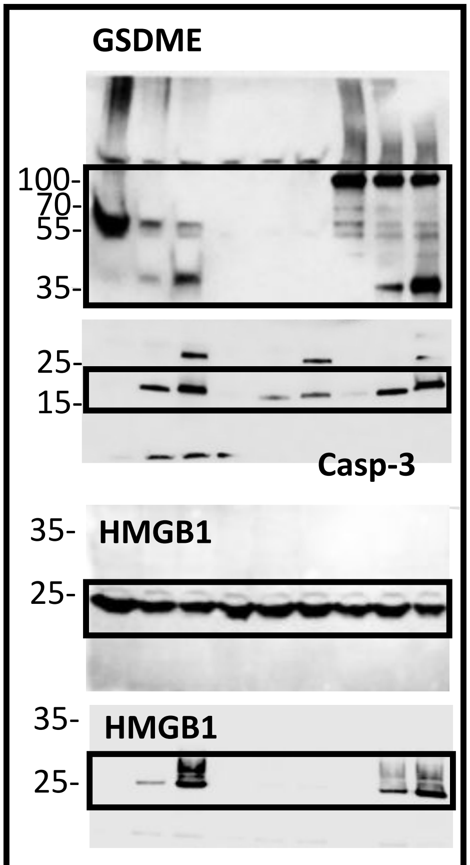

Sup. Fig. 2

Supplementary Figure 28. Uncropped scans of western blots shown in Sup. Figs. 1-2.

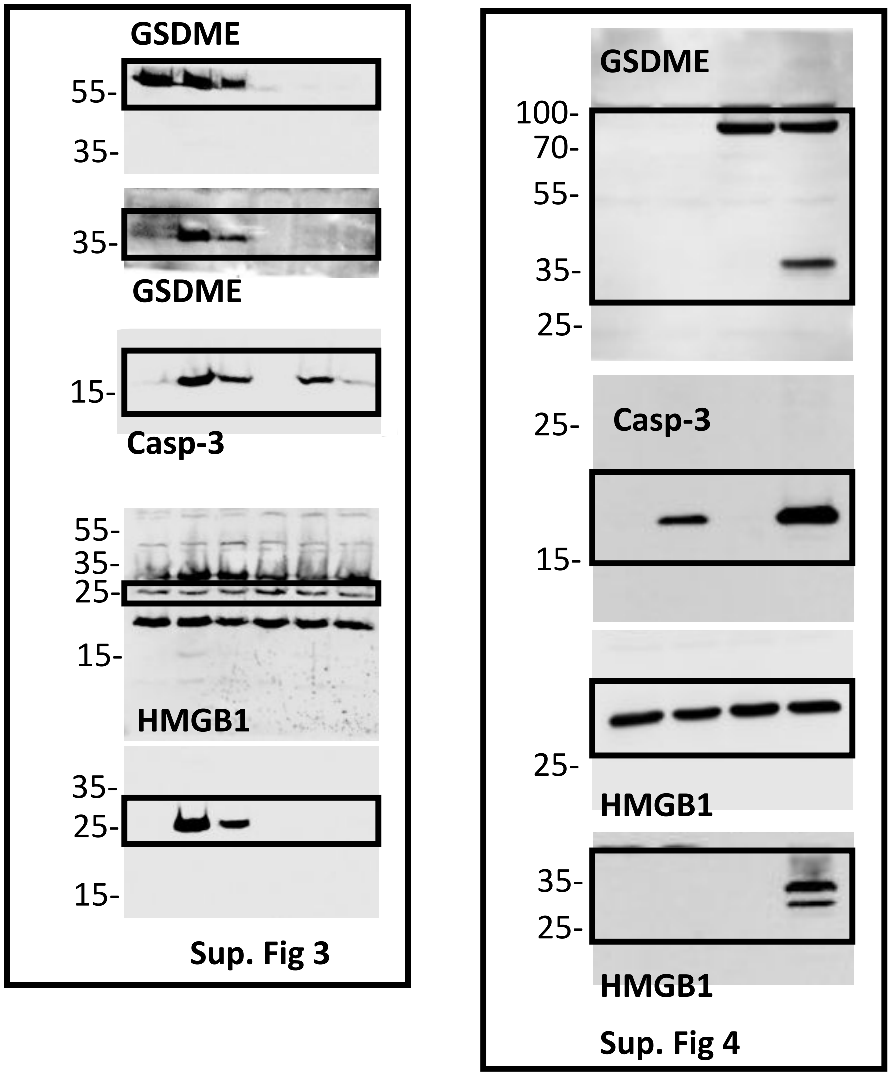

Supplementary Figure 29. Uncropped scans of western blots shown in Sup. Figs. 3-4.

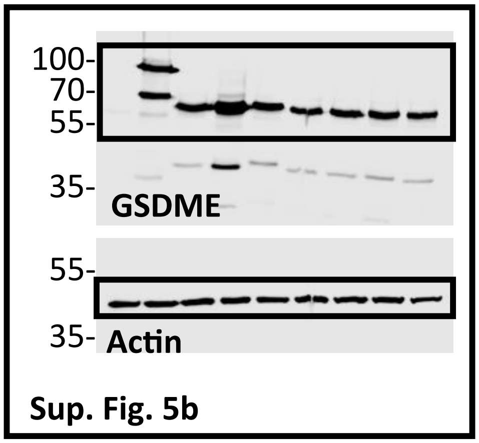

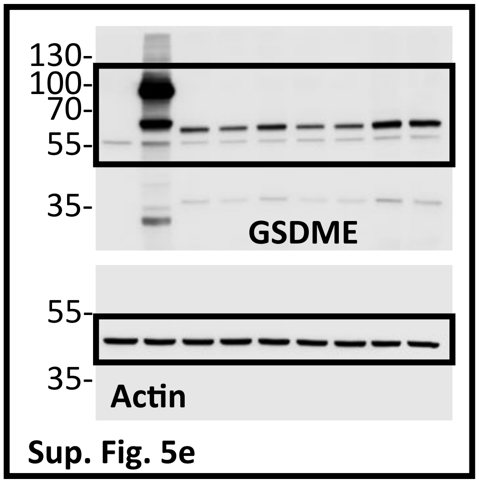

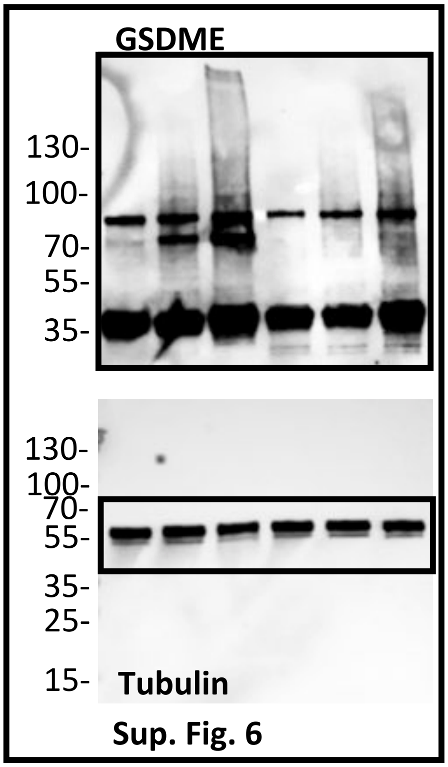

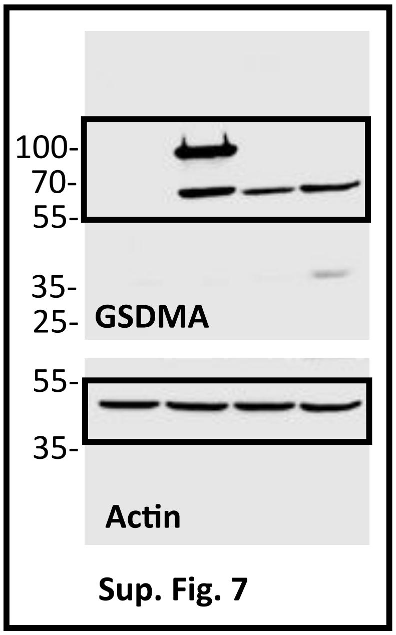

Supplementary Figure 30. Uncropped scans of western blots shown in Sup. Figs. 5-7.

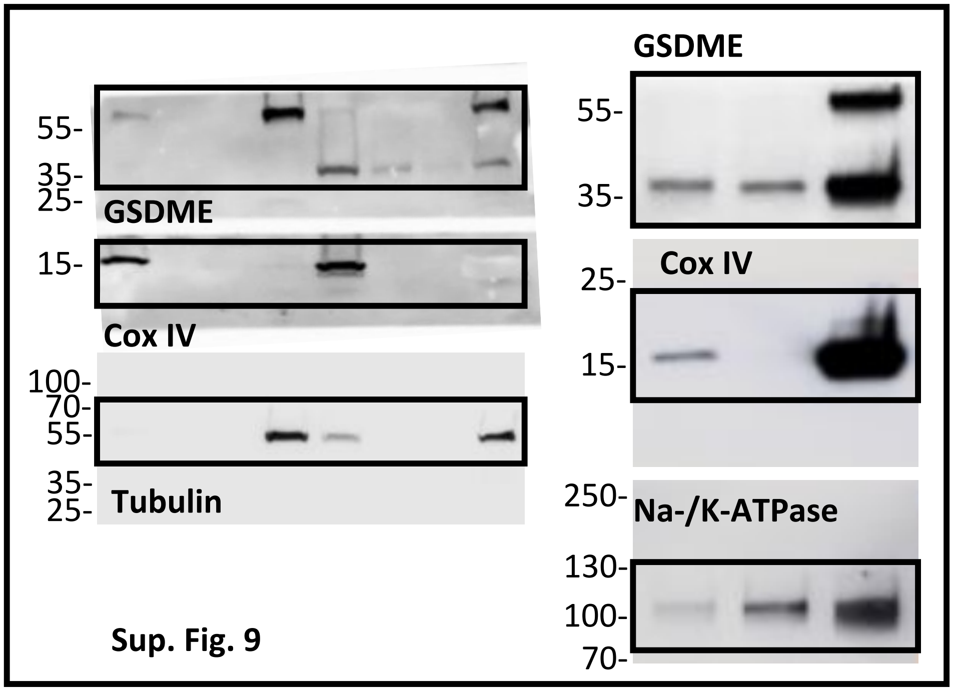

Supplementary Figure 31. Uncropped scans of western blots shown in Sup. Figs. 9.

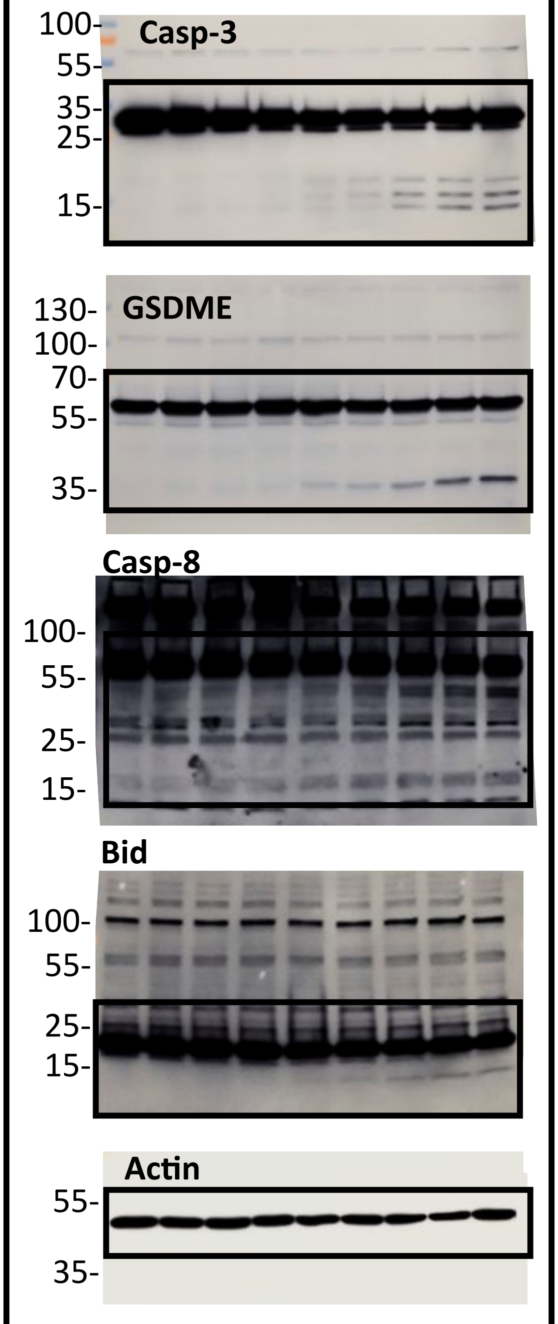

Sup. Fig. 11

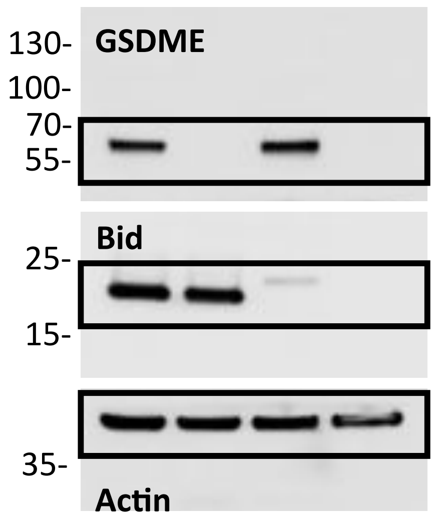

Sup. Fig. 12

Supplementary Figure 32. Uncropped scans of western blots shown in Sup. Figs. 11-12.

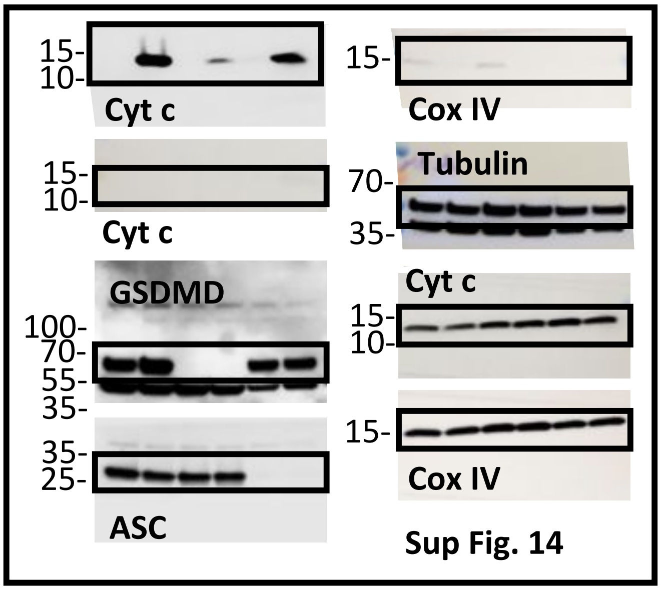

Supplementary Figure 33. Uncropped scans of western blots shown in Sup. Figs. 14.
